# Practical considerations for birefringence microscopy of myelin structure: Microscope design and tissue processing for effective imaging

**DOI:** 10.1162/imag_a_00186

**Published:** 2024-06-03

**Authors:** Nathan Blanke, Alexander J. Gray, Rhiannon E. Robinson, Anna Novoseltseva, Douglas L. Rosene, Irving J. Bigio

**Affiliations:** Department of Biomedical Engineering, Boston University, Boston, MA, United States; Department of Anatomy and Neurobiology, Boston University School of Medicine, Boston, MA, United States; Department of Electrical and Computer Engineering, Boston University, Boston, MA, United States

**Keywords:** birefringence microscopy, sample preparation, label-free, myelin, quantitative imaging

## Abstract

Despite the interest in studying and quantifying the structural integrity of myelin in postmortem brain tissue, current methods for high-resolution imaging of myelin with optical microscopy are not sufficient. While imaging methods must have adequate resolution and sensitivity to detect microstructural alterations to myelin that are relevant in aging and neurodegenerative disease, an equally critical aspect is to minimize myelin damage that is induced during tissue processing steps. Birefringence microscopy (BRM) is a powerful technique that leverages the structural anisotropy of myelin to provide detailed, label-free images of myelin at any diffraction-limited optical resolution, while maintaining a simple and low-cost setup. Building on our previous work, we have developed a new BRM system and image processing pipeline that enable efficient, high-throughput imaging of myelin structure at multiple scales. Here, we utilize this system to systematically assess the damage to myelin that is induced by several common tissue processing steps in brain sections from the rhesus monkey. Images taken of the same myelinated axons, before and after each tissue processing step, provide direct evidence that mishandling of tissue during sample preparation can cause significant structural alterations to myelin. First, we report on key advancements to our BRM system, imaging procedure, and image processing pipeline, which provide significant increases to the speed and efficiency of BRM. These include integrating fast piezoelectric rotational stages, minimizing the number of images required (to three images) for determining birefringence parameter maps, and implementing an analytical solution for directly determining birefringence parameter maps. Second, using this BRM system, we demonstrate that effective myelin imaging requires (1) the avoidance of prolonged drying or dehydration of tissue, (2) the selection of the optimal mounting medium (85% glycerol), (3) the avoidance of tissue permeabilization with detergents (i.e., Triton X-100 and Saponin), and (4) the selection of a suitable tissue-section thickness (15, 30 and 60 µm) based on the region of interest. In addition to serving as a guide for new users interested in imaging myelin, these basic experiments in sample preparation highlight that BRM is very sensitive to changes in the underlying lipid structure of myelin and suggest that optimized BRM can enable new studies of myelin breakdown in disease. In this work, we show that BRM is a leading method for detailed imaging and characterization of myelin, and we provide direct evidence that the structure of myelin is highly sensitive to damage during inadequate preparation of brain tissue for imaging, which has previously not been properly characterized for birefringence imaging of myelin. For the most effective, high-resolution imaging of myelin structure, tissue processing should be kept to a minimum, with sections prevented from dehydration and mounted in 85% glycerol. With proper preservation of myelin structure, BRM provides exquisitely detailed images that facilitate the assessment of myelin pathology associated with injury or disease.

## 1 Introduction

Myelin is fundamental for efficient communication within the brain and provides axons with essential support throughout development (Nave & Werner, 2014;Saab & Nave, 2017;Stadelmann et al., 2019). In the aging or diseased brain, damage to myelin structure likely plays a major role in the progressive loss of cognitive and motor function (Bartzokis, 2004;Peters & Rosene, 2003). For postmortem characterization of myelin, electron microscopy (EM) has long been the gold-standard technique, as it enables detailed imaging of myelin ultrastructure (Bowley et al., 2010;Matsushima & Morell, 2001;Peters, 2009;Peters & Sethares, 2002). While EM provides unmatched nanoscopic resolution, factors such as preparation time, cost, and the very limited field-of-view render it impractical for quantifying myelin pathology over larger tissue volumes. Light microscopic approaches provide larger field-of-view and technical ease, but current techniques often face limitations when the goal is to study the structural integrity of myelin—not simply its presence. While EM has remained*the*method for imaging individual myelinated axons, other forms of microscopy have been slow to offer alternatives for imaging myelin structure across larger spatial scales.

With microscopy, myelin imaging can be performed at the level of individual myelinated axons or of bundles of myelinated axons (i.e., white matter). The most traditional method for imaging myelin is by staining myelin lipids with chemical compounds or dyes (Kiernan, 2007) and viewing the stained tissue with a standard light microscope. Conventional myelin staining informs on the spatial distribution and density of myelin but is limited for characterizing myelin structure, especially at high resolution. In more modern approaches, high-resolution, depth-sectioned images of myelin can be obtained with confocal microscopy and fluorescent labeling techniques, enabling myelin imaging at the single-axon level. Fluorescent labeling of myelin is carried out with either immunohistochemistry (IHC) to the major structural proteins of myelin, myelin basic protein (MBP) or proteolipid protein (PLP) (Barker et al., 2013;Kuhlmann et al., 2017;Mollink et al., 2017), or by staining with a fluorescent lipophilic dye (Budde & Frank, 2012;Monsma & Brown, 2012). Given its labeling specificity to myelin proteins, IHC seems like an obvious choice for high-resolution imaging. However, as tissue preparation for IHC requires full or partial delipidation of membranes to achieve efficient labeling of myelin proteins, the lipid structure of myelin itself is degraded by the labeling process. As such, IHC labeling of myelin proteins does not enable imaging of the structural integrity of the myelin sheath and is instead reserved for more general imaging of myelin spatial distributions or for providing colocalization with other structures, such as labeled cells or proteins.

Alternatively, high-resolution confocal imaging of myelin can be performed with fluorescent lipophilic dyes (Budde & Frank, 2012;Monsma & Brown, 2012), which directly stain myelin lipids. Although fluorescent staining of lipids is not specific to myelin, the multiple layers of lipid present in the myelin sheath provide higher contrast than the cells and other structures with single lipid bilayers. Most importantly, fluorescent staining does not require additional tissue processing steps, such as permeabilization; thus, it preserves the structure of myelin for imaging and characterization. While fluorescent labeling techniques provide familiarity and make use of advancements in confocal microscopy, these approaches are generally time consuming and invoke expensive instrumentation, limiting their use for routine myelin imaging or for imaging large volumes of tissue.

As evaluation of disease-related changes in myelin integrity necessitates imaging of individual axons, maintaining myelin microstructure during handling and processing of tissue is critical. Label-free approaches have the advantage of imaging based on endogenous contrast, which enables assessment of brain tissues while they are as close as possible to their in vivo state. Various forms of label-free optical microscopy have been demonstrated for reflectance imaging of myelin (coherent anti-Stokes Raman scattering [CARS] (Canta et al., 2016;Gasecka et al., 2017;Ozsvár et al., 2018;Wang et al., 2005), third-harmonic generation (Farrar et al., 2011), spectral confocal reflectance [SCoRe] (Hill et al., 2018;Schain et al., 2014), and two-photon autofluorescence (Costantini et al., 2021;Sorelli et al., 2023)); however, similar to confocal fluorescence imaging, these techniques require point-scanning and are ultimately inefficient for imaging across larger volumes of tissue.

For more flexible, label-free imaging, there are simpler*widefield*(camera-based) methods that leverage the unique optical properties of myelin, enabling myelin to be viewed directly by the user or camera during imaging. Due to the compact, multilamellar structure of the myelin sheath, it exhibits a high degree of structural anisotropy. This structural anisotropy of myelin gives rise to*optical birefringence*(Bear et al., 1937;de Campos Vidal et al., 1980;Setterfield & Sutton, 1935), which is a material property that results in the optical refractive index being dependent on the orientation of light polarization. The optical birefringence of myelin presents an excellent opportunity for direct, structural imaging with polarized-light microscopy. Polarized-light microscopy of myelin has been understood for decades (Axer et al., 2001,^2011^;Guo et al., 2020;Schnabel, 1966), but it remains an area that is underutilized by neuroscientists. In particular, the powerful capabilities of*3D-polarized light imaging*(*3D-PLI*) have been demonstrated byAxer et al. (2011)for mapping directionality and connectivity of white matter tracts in the human brain, but has not been adopted for high-resolution imaging of myelinated axons and myelin pathology. For those studying disease with polarized-light imaging techniques (Mollink et al., 2019;Morgan et al., 2021), investigations have only been carried out at low resolution, above the level of individual axons. In our work, focused on evaluating myelin degradation in postmortem brain sections, we perform rapid and detailed birefringence imaging down to the level of individual myelinated axons, with the technique we have chosen to refer to as*birefringence microscopy*(BRM) (Blanke et al., 2021).

Motivated by our own studies in the rhesus monkey brain, we have focused our efforts on optimizing methods for high-throughput, label-free BRM. Our goal is to facilitate quantifying the subtle (or not-so-subtle) changes in myelin structure that occur in aging, injury, and disease; and for these applications, preservation of myelin structure during all steps of tissue handling and slide preparation is imperative. Alterations to myelin structure induced by common sample preparation methods have not been adequately characterized for birefringence imaging of myelin. Using BRM, we have established that myelin is highly sensitive to damage caused by common sample preparation techniques and have utilized this information to arrive at an optimized protocol for structural imaging of myelin. Here, we describe the design of a high-throughput BRM system and an optimized image processing pipeline for efficient, quantitative imaging of myelin structure at multiple scales. We utilize this system to evaluate four factors in the preparation of tissue sections that are vital in enabling high-resolution, structural imaging of myelin with BRM: (1) preventing the tissue from drying on the microscope slide, (2) selecting the optimal mounting medium (85% glycerol), (3) avoiding detergent permeabilization, and (4) selecting a suitable tissue-section thickness based on the region of interest. Furthermore, these results demonstrate both that myelin is sensitive to breakdown from common sample preparation protocols and that BRM is well suited for characterization of microscopic structural changes that would otherwise require considerably more time and resources with other techniques. With adequate preservation of myelin integrity during preparation of brain tissue, BRM can provide exquisitely detailed images of individual myelinated axons and their microscopic structural properties, with diffraction-limited optical resolution up to ~250 nm. This enables myelinated axons to be studied in new contexts and facilitates more reliable and quantitative assessment of myelin pathology.

## 2 Methods

### 2.1 Sample preparation

#### 2.1.1 Tissue section preparation

A normal aging rhesus macaque monkey (age: 20.9) was anesthetized and exsanguinated during transcardial perfusion with ice-cold Krebs buffer at 4°C to clear vasculature and halt cellular deterioration. This was immediately followed by perfusion with 37°C 4% paraformaldehyde to fix the brain. Perfusion of fixative at body temperature (37°C) is done to speed the actual fixation to reduce time for autolysis. Although we have not examined temperature systematically, there are known advantages and disadvantages for various temperatures for fixation (McFadden et al., 2019). In prior work, fixation at 37°C has been successful for immunohistochemical studies (Shobin et al., 2017) as well as for electron microscopic studies (Ngwenya et al., 2005,^2008^). In our experience, fixation at this temperature works well for imaging of myelin. The brain was blocked in situ in the coronal plane and immersed overnight in 4% paraformaldehyde at 4°C. The brain was then removed from fixative, rinsed in buffer, and immersed in a series of 10–20% glycerol + 2% dimethylsulfoxide (DMSO) baths for cryoprotection. Slabs or pieces were divided from the block and were flash-frozen at -75°C in isopentane and stored at -80°C until being cut into 15-, 30-, and 60-µm-thick frozen sections, which were then stored in 0.05 M Tris-buffered saline (TBS) with 15% glycerol for cryoprotection. This cryoprotection protocol has demonstrated superb immunohistochemical reaction for myelin imaging, therefore, we are confident that sections imaged with BRM are not confounded by any unbound fixative or rehydration of the tissue in buffer (Estrada et al., 2017). For all experiments, sections were taken from only one monkey to limit variability in tissue quality. As a technical note, frozen sections are used for BRM, as paraffin embedding is incompatible due to the chemicals that are required for deparaffinization (e.g., xylene), which specifically dissolve lipids. Care of the animals used in this study was accredited by the Association for the Assessment and Accreditation of Laboratory Animal Care (AAALAC) and in accordance with the Guide for the Care and Use of Laboratory Animals from the National Institutes of Health’s Office of Laboratory Animal welfare and approved by the Institutional Animal Care and Use Committee (IACUC) of Boston University Medical Campus.

#### 2.1.2 Slide preparation

Following our established protocol for BRM, vials of rhesus monkey brain sections are removed from the freezer, thawed rapidly at room temperature, and transferred from their vial to a shallow dish filled with either 0.05 M TBS or phosphate-buffered saline (PBS). The sections are gently washed for 15–30 minutes (depending on section thickness) to wash away the cryoprotectant (15% glycerol). The tissue is then carefully guided onto a submerged gelatin-coated slide with a fine-tipped paintbrush and held down by the paintbrush while raising the slide from the dish of buffer solution. Excess solution is drained and blotted from the slide, while using the paintbrush to minimize folding or wrinkling of the tissue. The remaining excess fluid is then quickly removed with lint-free optical grade lens paper (Edmund Optics, #52-105) by applying several dry pieces to the edges of the slide and directly on top of the tissue. After removal of excess fluid, care is taken to minimize the amount of time that the section is allowed to air dry, so that as soon as it adheres to the microscope slide, it is mounted in 85% glycerol (15% deionized H_2_O, 0.01% sodium azide) and coverslipped to preserve myelin structure and match the refractive index (RI) of myelin lipids (n ≈ 1.46) (Pusterla et al., 2017). Paper towels are used to dry excess glycerol from the edges of the slide, and nail polish is used to completely seal the coverslip to the slide. All baseline or control images in this paper were taken from sections mounted according to this protocol.

#### 2.1.3 Experimental protocol: preserving myelin by preventing the tissue from drying on the microscope slide

Four brain sections were selected for assessing the effects of prolonged air drying or dehydration of the sample during the slide preparation process. In the prepared slides, several regions of interest (ROIs) in gray and white matter were imaged with BRM to acquire baseline images of myelinated axons before assessing damage due to prolonged drying. After acquiring baseline images, the coverslip was removed with a razor blade and the samples were all washed with buffer solution (0.05 M PBS, to avoid salt residue from being left on the slide) to remove the 85% glycerol mounting medium. Slides were then removed from the buffer solution and optical lens paper was used to remove excess solution from the slide and tissue sample (seeSec. 2.1.2). Samples were then allowed to air dry for an additional 0 minutes (control), 15 minutes, 1 hour, or 24 hours. For the control sample (0 minutes drying), the coverslip was removed, the sample was washed in buffer, dried of excess fluid, and was then immediately mounted in 85% glycerol once again and re-coverslipped. All other samples underwent the same procedure but following the washing step, and after being dried of excess fluid, the sample was allowed to further air dry for the corresponding time at room temperature. After this drying period, samples were mounted with 85% glycerol once again and re-coverslipped as described above. BRM images were acquired in the same ROIs before and after this experimental drying for direct visualization of any damage induced to myelin. A subset of these slides were compared with others that were given an additional rinse of dH_2_O before being left to dry to confirm that the buffer solution used does not cause crystallization damage.

#### 2.1.4 Experimental protocol: preserving myelin and refractive index-matching by selecting the correct mounting medium

Two brain sections were used to compare the degree of sample preservation and RI-matching provided by two different mounting media, 2,2’-thiodiethanol (TDE) and PBS. TDE is a common optical clearing agent and RI-matching mounting medium used for imaging in brain tissue (Costantini et al., 2015;Staudt et al., 2007), while PBS is a generic aqueous mounting medium (n = 1.33) that serves as a control in this experiment. These two mounting media, which are not suitable for BRM, were chosen to demonstrate the two main concepts important for imaging of myelin birefringence: preservation of myelin structure and RI-matching. Both sections were initially prepared as described inSec. 2.1.2, and baseline BRM images were acquired from several gray and white matter regions while the sample was mounted in 85% glycerol. After baseline imaging, the coverslips were removed, the 85% glycerol mounting medium was rinsed away, and the sections were prepared for imaging in another mounting medium. The first section was processed for TDE clearing and mounting by immersion in 30% TDE for 15 minutes, followed by a final immersion in 60% TDE for 15 minutes (Aoyagi et al., 2015) to show a loss of myelin structure at the expense of RI-matching through optical clearing. The second section was simply mounted in PBS to simulate poor RI-matching while preserving myelin structure (and serving as a control). For direct comparison, BRM images were acquired from the same ROIs as before, when the sections were mounted in 85% glycerol. After imaging the TDE-mounted and PBS-mounted sections, the coverslips were once again removed, the experimental mounting medium was washed away, and the sections were mounted in 85% glycerol and re-coverslipped to assess if there was permanent damage to myelin.

#### 2.1.5 Experimental protocol: preserving myelin by avoiding detergents used for membrane permeabilization

Ten brain sections were selected for demonstrating the adverse effects of permeabilization with detergents, commonly used in IHC protocols for antibody labeling of frozen sections. The samples were initially mounted following the protocol inSec. 2.1.2and baseline images were acquired before damage to myelin. After baseline imaging, the coverslip was removed, and the sections were rinsed and subjected to different concentrations of Triton X-100 (TX-100). Individual tissue sections were washed with 0.1 M TBS (control), 0.1%, 0.5%, 1.0%, and 1.5% TX-100 in 0.1 M TBS for 30 minutes at room temperature on a slow rocker. Two brain sections were imaged for each concentration. These concentrations of TX-100 span the range that are useful for different applications of IHC labeling in frozen sections. While lower concentrations (0.1–0.2%) are sufficient and typical for permeabilizing and labeling of cells (Koley & Bard, 2010;Mattei et al., 2017), we have found that IHC of MBP typically requires use of higher TX-100 concentrations (0.5%, 1.0%, or 1.5%) in order for the antibody to penetrate into myelin sheaths. Following washing with TX-100, the sections were left free floating in 0.1 M TBS for 2 hours at room temperature on a slow rocker. The tissue was then re-coverslipped and imaged at high resolution within the same ROIs to assess the structural damage to myelinated axons.

#### 2.1.6 Experimental protocol: Imaging details of individual axons by selecting an optimal tissue-section thickness

Three brain sections were cut at different thicknesses (15 µm, 30 µm, and 60 µm) from the preserved whole hemisphere of a rhesus monkey, within the same anatomical region of the brain. Each section was mounted according to the protocol inSec. 2.1.2, and several ROIs from dense white matter (corpus callosum) and more myelin-sparse gray matter were imaged with high-resolution BRM. The ability to clearly resolve the details of individual myelinated axons was assessed qualitatively, based on the combined effects of the section thickness and the density of myelinated axons in the ROI.

### 2.2 Birefringence microscopy (BRM)

**Fig. 1.**
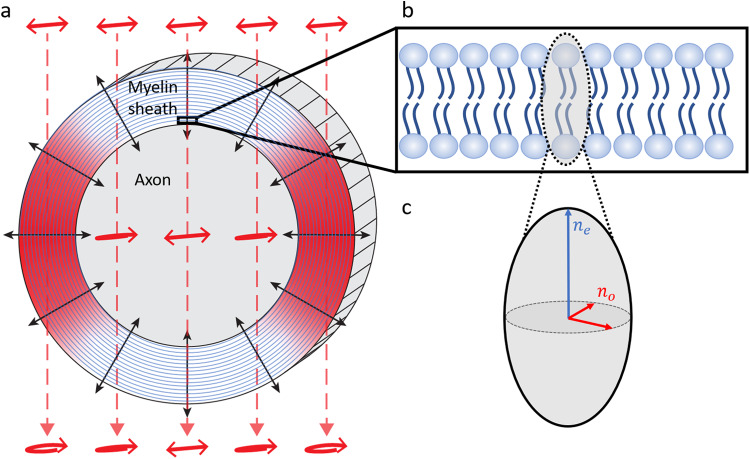
Optical birefringence of myelin. (a) Diagram of myelin birefringence for a myelinated axon oriented longitudinally within tissue (parallel to the section surface), with linearly polarized light incident from above. In this geometry, the edges of the multilayer myelin sheath (indicated by red shading) generate birefringence contrast, as those regions have an optic axis (indicated by black arrows) that has components orthogonal to the direction of light propagation (indicated by dashed red lines). In these regions, the incident linear polarization is altered by propagating through the birefringent medium and emerges elliptically polarized. For the top and bottom of the myelin sheath, where the optic axis is parallel to the direction of light propagation, polarized light passes through unaltered, and the myelin generates no birefringence contrast. (b) Expanded diagram of a single lipid bilayer of the myelin sheath. The direction of myelin anisotropy corresponds to the directionality of the lipid tails. (c) Diagram of the refractive index ellipsoid, overlaid on the lipid bilayer in (b). The direction of anisotropy is said to exhibit the extraordinary index of refraction,$n_{e}$, while the orthogonal directions exhibit the ordinary index of refraction,$n_{o}$.

BRM is a widefield (camera-based) imaging technique, in which label-free images of sample birefringence are generated by detecting changes to the polarization state of incident light. We have previously demonstrated BRM for imaging myelin structural breakdown following localized ischemic injury in the rhesus monkey (Blanke et al., 2021) and for validating anatomical fiber orientations in human brain tissue (Blanke et al., 2023). As compact myelin exhibits strong birefringence, its structural anisotropy can be readily imaged with BRM (Fig. 1a). The directionality of the aligned lipid molecules that make up the myelin sheath (Fig. 1b) results in an optic axis that is radial with respect to the longitudinal axis of its axon (Bear et al., 1937;de Campos Vidal et al., 1980) (shown with black arrows inFig. 1a). As a consequence of the radial optic axis of myelin, when performing BRM on myelinated axons oriented longitudinally within the tissue section (polarized light incident from above), only the edges of the myelin sheath will generate birefringence contrast (indicated by red shading inFig. 1a). The same is not the case for myelinated axons oriented transversely within the tissue section, as in this geometry, birefringence contrast will be exhibited along the full circular cross-section. During BRM, by illuminating the sample with polarized light and detecting changes to that input polarization state, the birefringence of myelin can be imaged qualitatively or quantitatively, dependent on the configuration of the microscope.

Our BRM system utilizes two complementary imaging modalities:*cross-circular-polarized*BRM (CCP-BRM) (Schnabel, 1966) for real-time imaging in extinction and*quantitative*BRM (qBRM) for rapid imaging of birefringence parameters (relative retardance and optic-axis orientation) (Axer et al., 2011;Glazer et al., 1996). CCP-BRM is a simple configuration achieved by imaging through crossed circular polarizers of opposite handedness on either side of the sample. CCP-BRM provides real-time contrast of any birefringent structure, regardless of optic-axis orientation, without the need to move any optical elements. As such, CCP-BRM enables direct, high-throughput imaging of myelin birefringence, where the imaging speed is primarily limited by the acquisition rate of the camera (exposure time limited) and the physical speed of the XYZ-scanning stages. Furthermore, CCP-BRM is a highly accessible imaging modality as a simple setup can be accomplished by inserting two off-the-shelf, low-profile circular polarizers (one left-handed and one right-handed) into the illumination and detection paths of virtually any commercial light microscope (seeSupplementary Note 2.3). For qBRM, the circular polarizer in the illumination arm is replaced with a linear polarizer, and the sample is sequentially illuminated at multiple linear polarization angles(θ). The transmitted light intensity,I(θ), is detected through a circular-polarization analyzer (by convention, the polarizing elements placed on the detection side of the microscope are referred to as the “analyzer”). Each pixel’s intensity variation at the detector, for each illumination-polarizer angle, can be modeled with Jones calculus, yielding the following equation:

$$I(\theta )=\frac{I_{0}}{2}\left[1+\sin(2(\theta -\varphi ))\cdot \sin(2\pi \delta )\right],$$ (1)

where the terms$I_{0}$,|sin(2πδ)|, andφrepresent the birefringence parameters of*transmittance, relative phase retardance*, and*in-plane optic-axis orientation*, respectively. These birefringence parameters can be solved by nonlinear fitting, discrete harmonic Fourier analysis (Axer et al., 2011;Glazer et al., 1996), or a direct mathematical solution (seeSec. 2.2.2). The relative retardance map informs on the density and degree of alignment of myelin within a pixel, while the optic-axis orientation map provides the directionality of the myelin axis of anisotropy. The transmittance map provides a measure of intensity lost due to scattering or absorption, but we note that absorption can typically be assumed to be negligible in thin, fixed brain sections. We typically do not analyze the transmittance image since we work to minimize scattering during slide preparation; however, transmittance (scattering) information can be used for correction or for estimating the out-of-plane orientation angles of fibers (Menzel et al., 2020,^2022^).

#### 2.2.1 Instrumentation for high-throughput BRM

**Fig. 2.**
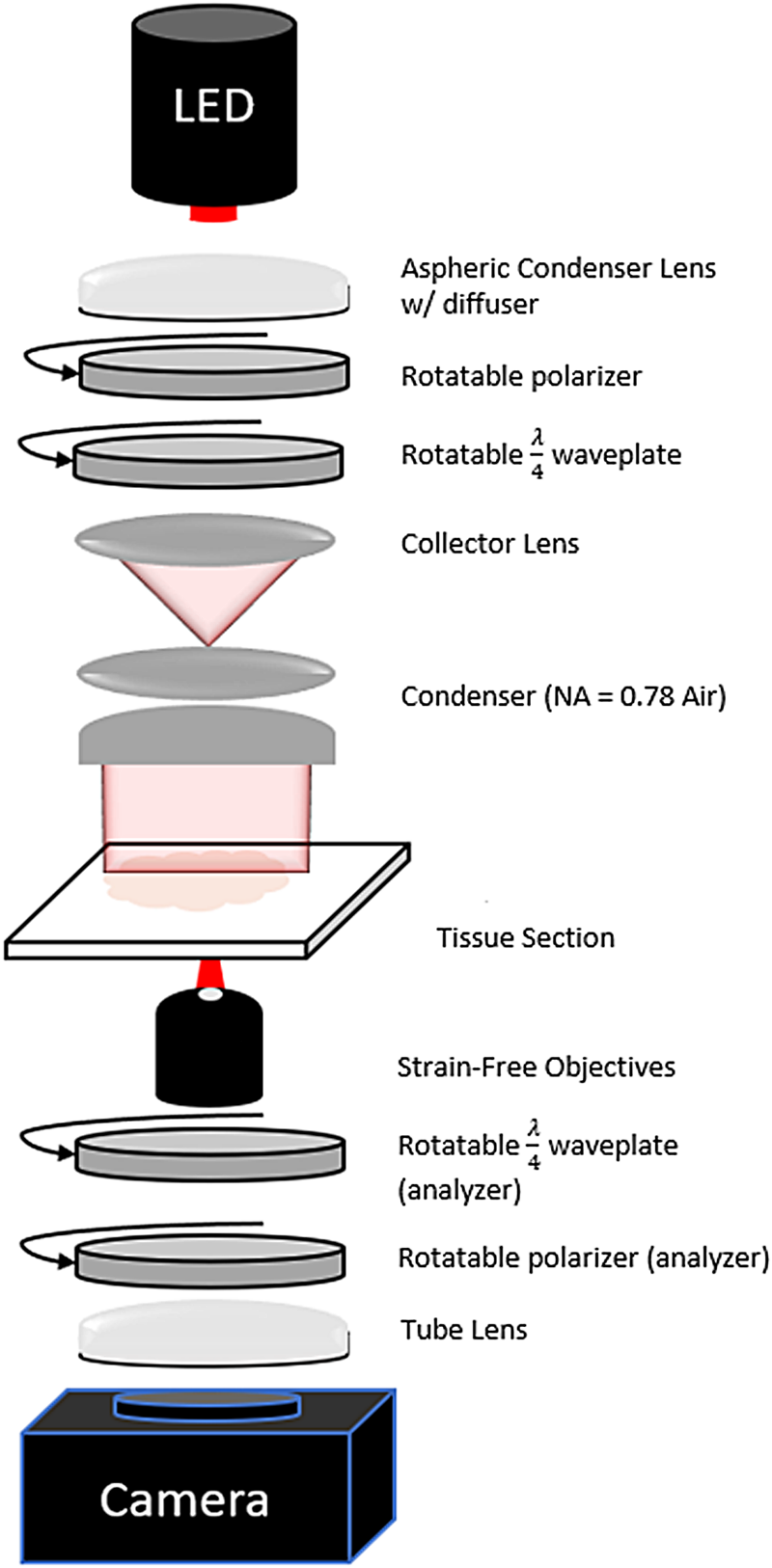
Optical schematic of high-throughput BRM system.

A new BRM system (Fig. 2) was designed based on our previous microscope (Blanke et al., 2021), enabling both CCP-BRM and qBRM of myelin across entire sections at submicron resolution with significantly increased speed and efficiency. The system was designed around the Thorlabs Cerna^®^Microscope Body to allow for full customization of our optical setup and to integrate high-speed stages for manipulating the optical polarization state. In addition to permitting faster acquisition of BRM images with hardware, a key improvement to our approach has been realized by minimizing the number of images needed for qBRM image analysis and by implementing analytical solutions for rapid determination of birefringence parameters (seeSec. 2.2.3). Within the new system, a high-power LED (Thorlabs M625L4, 625 nm) is used for illumination of the sample with narrowband light, and an aspheric condenser lens with a diffuser on the plano surface (Thorlabs ACL2520U-DG6-B) is used to approximately collimate the LED before going through the illumination-side polarization optics. Thin-film linear polarizers (Thorlabs LPVISE100-A) and polymer true zero-order quarter-wave plates (Thorlabs WPQ10ME-633) provide high extinction and flexible control of polarization ellipticity during imaging. The illumination-side polarizer and waveplate are used to illuminate the sample with either circularly polarized (CCP-BRM) or linearly polarized light (qBRM), while in the detection side of the microscope, the polarizer and quarter-wave plate pair are always used as a circular analyzer.

For rapid, volumetric image acquisition at any optical resolution, we utilize a fast XY-scanning stage (Thorlabs MLS203-1) and Z-scanning stage (Thorlabs ZFM2020), as well as strain-free objective lenses with a range of numerical aperture (NA) values (4X [NA 0.13], 10X [NA 0.3], 20X [NA 0.5], 40X [NA 0.75], and 60X oil immersion [NA 1.35]). We use strain-free objectives to minimize potential sources of residual system birefringence, but it should be noted that special objective lenses are not strictly required. After the objective and circular analyzer, a tube lens (Thorlabs TTL200-A) is used to transfer the image to the camera. The current system incorporates a high-speed, cooled, large field-of-view (FOV) camera (Teledyne-Iris 9, 9MP @ 16-bit, 55 FPS), which fully captures the FOV of the objective with sufficient pixel sampling. Custom MATLAB software is used to enable precise control of the motorized stages and acquisition parameters for various types of tiled and z-stacked image sets. As a technical note, other wavelength options are available for BRM (blue, green, or NIR), assuming that the corresponding quarter-wave plates are also purchased; however, there is always a tradeoff between wavelength-dependent optical scattering, optical resolution, and birefringence sensitivity.

In our new BRM system, all polarization optics are mounted on high-speed piezoelectric rotation stages (Thorlabs ELL14), allowing for rapid switching (430°/second) between different polarization states. High-speed switching from CCP-BRM to qBRM is accomplished by rotating the illumination linear polarizer to align its transmission axis to the fast (or slow) axis of the illumination waveplate, so that the combination produces linearly polarized light. The two are then synchronously rotated to acquire qBRM image sequences. This greatly increases practical imaging speed by facilitating efficient switching between real-time viewing of the sample with CCP-BRM and qBRM acquisition without removing any optical elements. For polarization control, we opted to use polymer true zero-order quarter-wave plates mounted in piezoelectric rotation stages, as opposed to electronically controlled liquid-crystal variable retarders (LCVRs), to maximize our extinction ratio and achieve higher contrast during imaging. Additionally, by using piezoelectric stages with high switching speeds, the difference in speed between mechanical rotation and electronic control (with LCVR-based systems) is significantly reduced. From a design standpoint, LCVRs also require bulky and expensive drivers for voltage and temperature control, which increase the complexity of the design and calibration. For users interested in BRM, there are generally three levels of implementation with different degrees of cost, complexity, and instrumentation requirements (seeSupplementary Note 2). As described here, a custom, fully automated BRM system can be built from scratch for <$45k, while if there is an existing light microscope available to be modified, similar performance can be achieved with the addition of polarization optics and motorized rotational stages for ~$2.5k. Furthermore, when modifying an existing microscope, a much simpler setup for only CCP-BRM can be realized for as low as $60.

#### 2.2.2 Multiscale myelin imaging with BRM

The optical resolution of our microscope can be approximated using Abbe’s diffraction limit, based on the NA of the objective lens and wavelength of light used for imaging. With red light (λ = 625 nm), our microscope provides a lateral resolution ranging from ~2.4 µm (4X objective, NA 0.13) to ~230 nm (60X objective, NA 1.35), allowing for imaging of even the smallest myelinated axons below 0.5 µm (Lamantia & Rakic, 1990;Liewald et al., 2014). When using lower-NA objectives (4X and 10X), the axial resolution of the objective is larger than (or approximately equal to) the thickness of the tissue section (typically ~30 µm), so additional structures are not resolved by imaging multiple focal planes (z-stacks) across the tissue. Instead, imaging of the tissue can be performed at a single in-focus z-plane. For higher-NA objectives (20X, 40X, and 60X), the axial resolution is typically smaller than the thickness of the tissue, therefore, z-stack acquisitions are needed to resolve structures coming into focus at different depths of the tissue section. By acquiring volumetric images of brain sections at high resolution, the organization of myelinated axons and their structural integrity can be studied in great detail. To adequately capture details along the thickness of the tissue, z-stack images are acquired at 2-µm, 1-µm, and 0.5-µm z-steps for the 20X, 40X, and 60X objectives, respectively.

#### 2.2.3 High-speed qBRM image analysis

Reconstruction of quantitative birefringence parameter maps requires measurements at multiple illumination–polarizer angles, followed by a computational approach to determine the phase (optic-axis orientation), amplitude (relative retardance), and DC-offset (transmittance) of the intensity sine wave (Eq. 1) exhibited by each pixel. Traditionally, during quantitative birefringence imaging, many polarization images (typically ≥6) are taken and parameter extraction is performed by nonlinear fitting (Blanke et al., 2021) or by discrete harmonic Fourier analysis (Axer et al., 2011;Glazer et al., 1996). To facilitate significantly faster image acquisition and qBRM analysis, we have derived a mathematical solution for determination of birefringence parameter maps, which utilizes only three qBRM images (at three angles of the illuminating linear polarizer) and does not require fitting. Our method involves directly solving for the three birefringence parameters, thus providing a more rapid and efficient workflow. In short, to minimize the number of illumination angles required for extracting the parameter maps, we utilize illumination polarizer angles of 0°, 60°, and 120°, which result in the following analytical solutions toEq. 1(solved with MATLAB symbolic variables),

$$I_{0}=\frac{2}{3}\left(I_{1}+I_{2}+I_{3}\right)$$ (2)

$$\left|\sin\left(2\pi \delta \right)\right|=\frac{2\sqrt{{\left(I_{1}\right)}^{2}+{\left(I_{2}\right)}^{2}+{\left(I_{3}\right)}^{2}-I_{1}I_{2}-I_{1}I_{3}-I_{2}I_{3}}}{I_{1}+I_{2}+I_{3}}$$ (3)

$$\varphi =\tan^{-1}\;\;\left(\frac{\sqrt{3}\cdot \left(I_{2}-I_{3}\right)+2\sqrt{{\left(I_{1}\right)}^{2}+{\left(I_{2}\right)}^{2}+{\left(I_{3}\right)}^{2}-I_{1}I_{2}-I_{1}I_{3}-I_{2}I_{3}}}{-2I_{1}+I_{2}+I_{3}}\right),$$ (4)

where$I_{1}$,$I_{2}$, and$I_{3}$are the pixel intensities for angles 0°, 60°, and 120°, respectively. To our knowledge, this is the first demonstration of using fewer than four polarization angles for reconstruction of birefringence parameter maps. This method yields the same results as discrete harmonic Fourier analysis but can be expanded to unequally spaced polarization angles and can be solved for any combination of angles. The solutions forEqs. 2–4change with different combinations of angles (e.g., 0°, 45°, and 90°); however, the angles 0°, 60°, and 120° provide the most accurate determination of the three birefringence parameters. With this 3-point method, we have developed an automated qBRM analysis pipeline, parallelized to run on an NVIDIA RTXA4000 GPU, which has further increased our processing speed and the rendering of qBRM images. In addition to enabling more rapid determination of birefringence parameters, the use of three angles also enables a novel qualitative rendering of birefringence orientation and retardance without postprocessing (seeSec. 2.2.5).

#### 2.2.4 Subtraction of system birefringence

For accurate birefringence parameter extraction during qBRM, “system” birefringence must be accounted for and subtracted properly during image analysis. In our setup, system birefringence is mainly due to the use of a monochromatic quarter-wave plate with a narrowband LED, as the circular analyzer will act as an elliptical analyzer for noncenter wavelengths of the LED spectrum. During qBRM acquisition, a background set of images,$I_{bg}$, are taken at a location with no sample and are used to correct for system birefringence. The sinusoidal component of the system birefringence trace is subtracted from the sample birefringence trace, for each pixel, to create a background-corrected qBRM image set (Eq. 5).

$$I_{corr}=I_{sample}-\left(I_{bg}-mean\left(I_{bg}\right)\right).$$ (5)

The corrected images are used to solve for values of relative retardance, transmittance, and optic-axis orientation inEqs. 2–4.

#### 2.2.5 qBRM visualization

Traditionally, after qBRM analysis, the individual maps of the relative retardance,|sin(2πδ)|, and transmittance,$I_{0}$, can be viewed directly as grayscale images, while further image rendering is required to generate the quantitative retardance-weighted optic-axis orientation map used for color visualization. We employ two methods for visualizing qBRM orientation data.

##### 2.2.5.1 “Unprocessed” qualitative qBRM visualization

In addition to the traditional qBRM image rendering, we have developed a novel way to qualitatively view qBRM images with zero computational processing. Raw qBRM images taken at 0°, 60°, and 120° are saved as the R, G, and B channels, respectively, in .TIFF file format. Storing the images this way provides an interesting qualitative color visualization of sample birefringence, without the need for processing (seeFigs. 3band4b). The combination of the three images provides a uniform sampling of the sinusoidal signal described byEq. 1, such that the color of each pixel is based on the optic-axis orientation, and the intensity of the color is determined by the relative transmittance and retardance (seeSupplementary Note 1). This provides a qualitative orientation map that can be used for real-time viewing of birefringent structures without requiring computational analysis. As a side note, this qualitative method for visualization of qBRM data does not default to the same colormap as the optic-axis orientation map that is rendered based on the orientation color wheel (see below).

##### 2.2.5.2 Retardance-weighted optic-axis orientation maps

Optic-axis orientation maps are rendered using a custom color wheel and colormap, where each pixel is assigned an RGB value based on the extracted in-plane optic-axis orientation. When displaying optic-axis orientation maps, a color-wheel scale provides visualization of the orientation data. Additionally, in the color image, the intensity of each pixel is then determined by its corresponding relative retardance value (retardance weighting). Retardance weighting of the optic-axis orientation map serves to automatically “mask out” the orientation information of pixels that do not contain birefringent structures, providing a straightforward method for visualization of retardance and orientation in one image.

#### 2.2.6 Interpreting qBRM parameter maps

The proper interpretation of qBRM parameter maps is important for effectively utilizing qBRM at different optical resolutions. The birefringence parameter maps, relative retardance and optic-axis orientation, are highly dependent on the thickness of the tissue section, the NA (and, consequently, the confocal parameter) of the objective lens, as well as the local orientation, in-plane and out-of-plane, and density of myelinated axons within the ROI. In some contexts, it makes sense to normalize retardance by the thickness of the tissue to estimate myelin quantity, but it is important to note that this assumes that relative retardance accumulates linearly (while propagating through a medium of uniform density and orientation), which is generally not true when comparing regions of tissue with complex fiber orientations.

In reality, many regions of the brain are composed of a heterogeneous mixture of fibers that do not equally contribute to the measured relative retardance value of each pixel. For example, the observed retardance will be lower in regions of the brain with crossing fibers or inclined (out-of-plane) fibers (Axer et al., 2011), due to the cancellation of relative phase delay of the polarization states of light that have propagated through variously (including orthogonally) oriented myelin optic axes. As a further complication, depending on the NA of the objective lens used during qBRM, this cancellation effect can be more or less pronounced. At low NA, there is a longer confocal parameter, which presents a greater pathlength through the tissue over which net relative retardance has an opportunity to cancel. At high NA, on the other hand, there is a short confocal parameter, which enables relative retardance images of individual myelinated axons with minimal addition or cancellation from fibers outside the imaging plane. It is important to note that, at the focal plane, all rays pass through the structures being resolved, while outside the focal plane, only a small fraction of the rays will pass through the out-of-focus structures. As a result, the structures near the focal plane contribute more significantly to the measured retardance than structures outside of the focal plane in the tissue. When imaging is performed at high NA, this results in high-contrast images that appear to be both darkfield and sectioned. In summary, the quantification of relative retardance (and the corresponding optic-axis orientation) across different brain regions or subjects is a nontrivial task that requires proper controls and understanding of the underlying contrast mechanisms of BRM. For the purposes of this study, given that we quantified the relative changes in retardance within the same ROIs, before and after each experimental step, it is reasonable to directly compare the mean relative retardance values between the two images.

#### 2.2.7 Focus stacking

For visualization of high-resolution myelin structure in 2D images (acquired at 20X, 40X, or 60X), volumetric images (z-stacks) are acquired across the full thickness of the section and are focus-stacked with Adobe Photoshop (seeSupplementary Note 4). Focus stacking is commonly applied in photography but can also be applied to microscopy z-stack images (Chen & Zhang, 2023;Forster et al., 2004). The Adobe Photoshop focus stacking algorithm is proprietary, but in essence works by selecting the most in-focus plane for each pixel and recombining the selected pixels into one 2D image, allowing for approximate viewing of all in-focus structures from multiple focal planes and minimizing storage needed for volumetric data. Focus stacking is used for visualization of all z-stack images acquired at high resolution in this work.

#### 2.2.8 Quantifying changes in relative retardance

As described above, BRM provides insight into the loss of structurally intact lipids that make up the myelin sheath and can be quantified by examining changes in the relative retardance across tissue sections. For the experiments shown inSec. 3.2–3.4of this work, relative retardance was quantified and compared, before and after each case of experimentally induced myelin damage, by taking the mean relative retardance in ROIs of the image which containing myelinated axons.

## 3 Results

### 3.1 High-throughput BRM

BRM enables flexible and detailed characterization of myelin structure in thin (<100 µm), fixed brain sections at all diffraction-limited optical resolutions. Depending on the application or the goals of the user, BRM can be employed qualitatively with CCP-BRM or quantitatively with qBRM (seeSec. 2.2). As described inSec. 2.2.1, we have optimized our system to enable fast switching between polarization states during image acquisition, while maintaining a relatively simple and cost-effective design, and have implemented a processing pipeline that effectively minimizes the number of images required (3 polarization images) for reconstruction of birefringence parameter maps. As expected, the accuracy when solving for the three birefringence parameters slightly degrades when solving with only 3 points. When comparing our 3-point method to sinusoidal fitting of the same measurement with 18 points, we observed a mean error of 1.18% for relative retardance and an average difference of 3.53° for optic-axis orientation. This analysis was only performed in regions of the tissue that exhibited high retardance signal (e.g., myelinated axons), as the error metrics become over-amplified in low-retardance regions (e.g., background). For analyzing specimens with strong birefringence, the 3-point method is effective and can be utilized to significantly reduce acquisition time and data storage. For applications that require imaging weakly birefringent specimens, more images will likely be required for the most accurate reconstruction of birefringence parameters. With our BRM system, we can image a 30-plane z-stack with CCP-BRM in ~5 seconds and with qBRM in ~24 seconds. By then tiling multiple z-stacks together, we achieve extremely rapid, volumetric imaging of myelin across large brain tissues, at a range of optical resolutions.

**Fig. 3.**
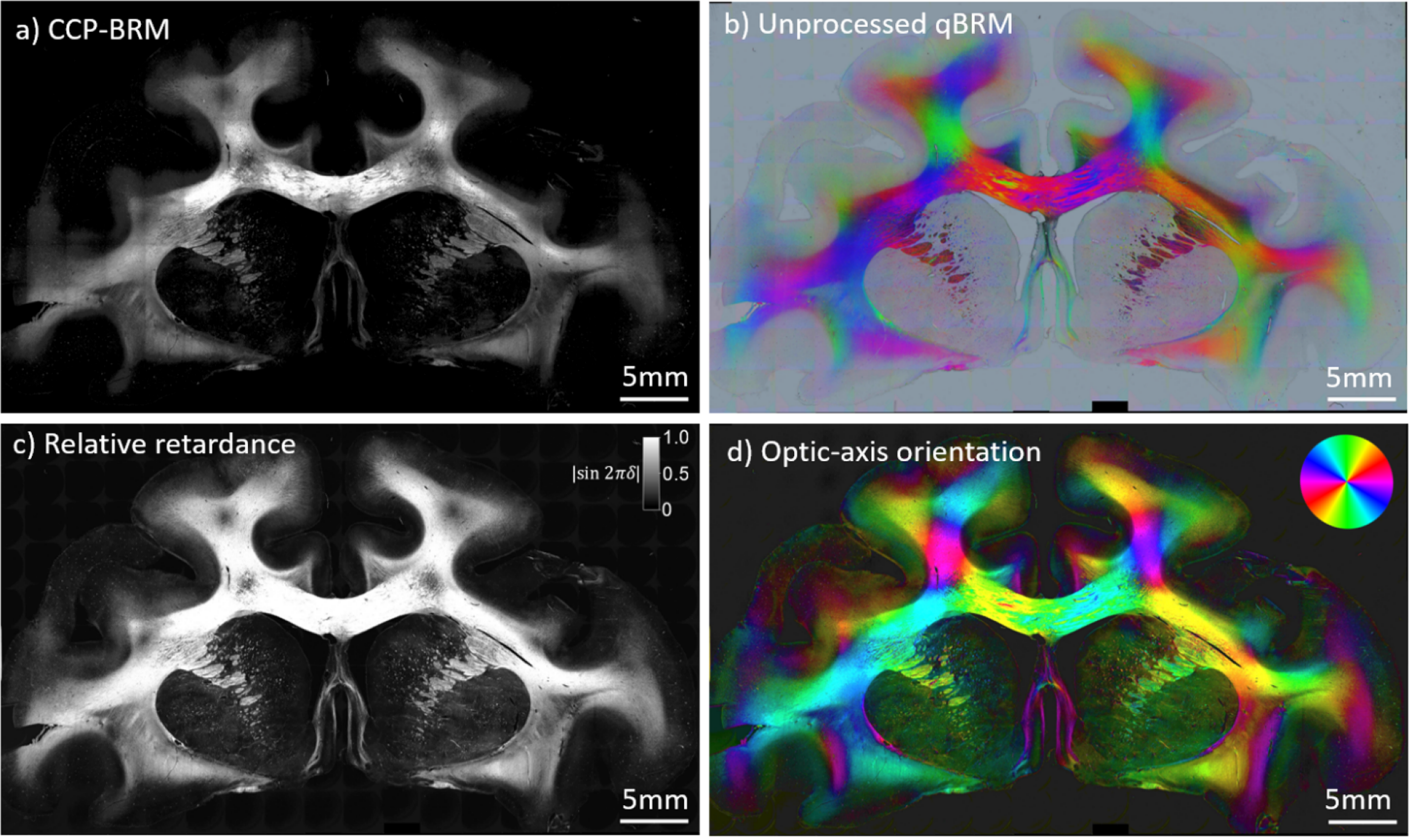
Low-resolution (4X, NA 0.13) tiled image set of an aged rhesus monkey coronal brain section from a cortical injury study [48],[49] visualized with different BRM techniques. (a) CCP-BRM. (b) Raw/unprocessed qBRM image data saved in RGB tiff file format. The qBRM images taken at 0°, 60°, and 120° are saved into the red, green, and blue channels of the RGB file, respectively, which creates a qualitative visualization of the retardance, optic-axis orientation, and transmittance (seeSupplementary Note 1). (c) qBRM relative retardance map. (d) qBRM retardance-weighted optic-axis orientation map. The color wheel in the top-right corner indicates the direction of the myelin optic axis, which is normal to the direction of the axonal trajectory.

**Fig. 4.**
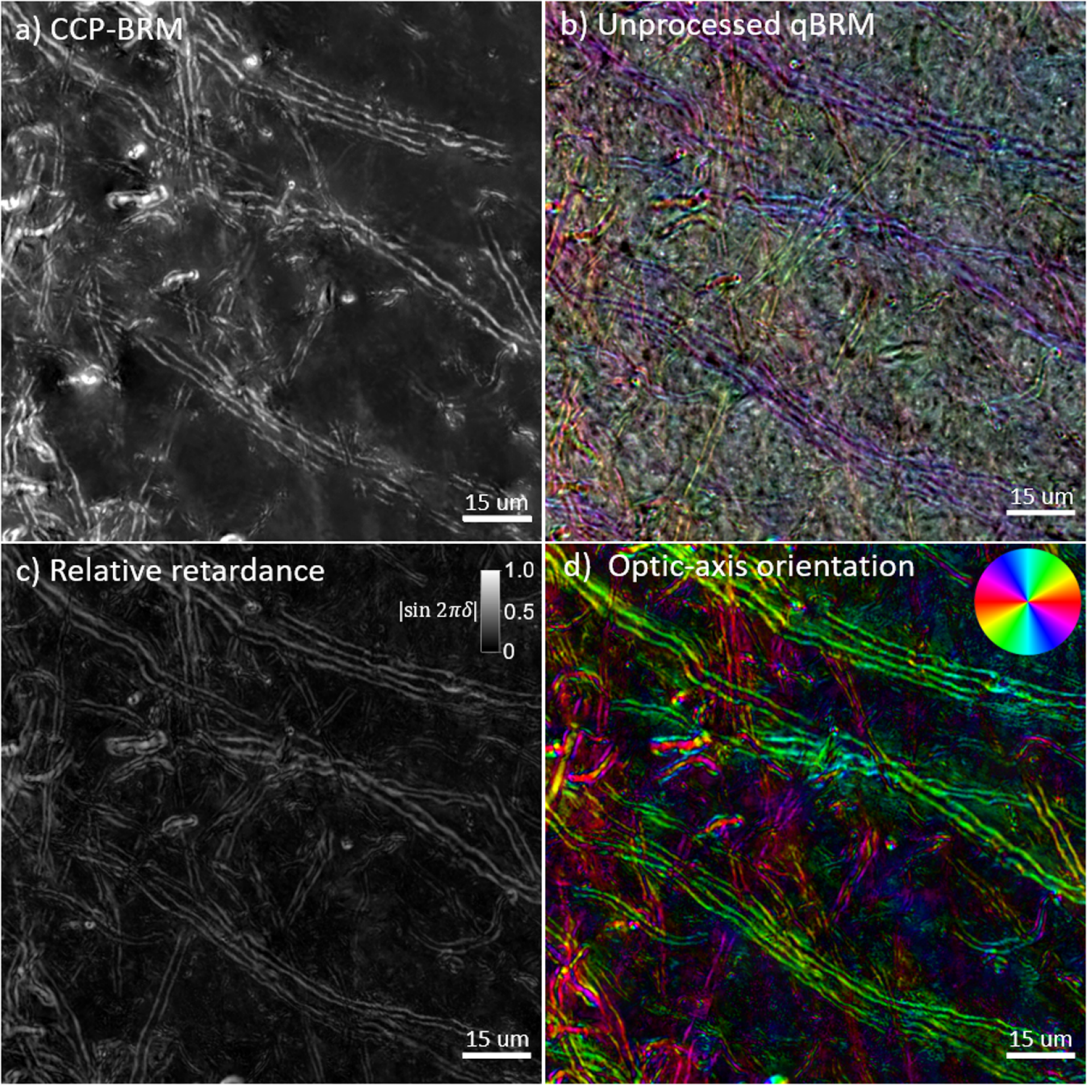
High-resolution (40X, NA 0.75) z-stack images (focus stacked) from a coronal rhesus monkey brain section in a transition region between white matter and gray matter with different BRM techniques. (a) CCP-BRM. (b) Raw/unprocessed qBRM image data saved in RGB tiff file format. The qBRM images taken at 0°, 60°, and 120° are saved into the red, green, and blue channels of the RGB file, respectively, which creates a qualitative visualization of the retardance, optic-axis orientation, and transmittance (seeSupplementary Note 1). (c) qBRM relative retardance map. (d) qBRM retardance-weighted optic-axis orientation map.

In a typical imaging session, the entire sample (ranging anywhere from ~1 to 12 cm^2^) is tiled and stitched at 4X magnification with CCP-BRM and qBRM (Fig. 3), then a region of interest is selected by the user for high-resolution imaging (Fig. 4). Images are acquired of a whole rhesus monkey coronal brain section from a cortical injury study (Go et al., 2019;Moore et al., 2012) to demonstrate the ability to image entire brain sections with BRM. CCP-BRM is used to bring the sample into focus and to set up an acquisition grid (in XY and Z) that designates where each tile location resides within the tissue. After acquisition, images are background corrected and stitched using the FIJI stitching plugin (Preibisch et al., 2009) with 10% overlap and linear blending. The low-resolution images provide a macroscopic view of the sample in one plane and can be used for analyzing the principal fiber orientations in white matter or assessing large-scale changes in myelin density in different disease models (e.g.,Morgan et al., 2021). Since the orientation of the myelin optic axis is always normal (radial) to the direction of the axonal trajectory (Fig. 1), there is an apparent 90° discrepancy when macroscopically viewing the true optic-axis orientation map (Fig. 3d) and expecting the image to display a map of fiber projections. For visualization purposes, many users may choose to rotate the color wheel by 90° to map the axon direction, rather than the orientation of myelin anisotropy. When viewing myelinated axons in high-resolution optic-axis orientation maps, the radial optic axis of myelin is more intuitive and can be more easily appreciated.

High-resolution BRM images enable clear visualization of the myelin sheaths of individual axons. CCP-BRM (Fig. 4a) provides high-contrast imaging of myelin, exactly as it is seen in real time, while the qBRM images (Fig. 4b–d) allow for visualization of quantitative birefringence parameter maps related directly to the anisotropic structure of myelin. The unprocessed qBRM images (Fig. 4b) are shown as they are saved in RGB format, and although they are not quantitative and contain uncoupled information about optic-axis orientation, retardance and transmittance, they have utility for real-time imaging and visualization without requiring computational analysis for rendering (seeSec. 2.2.4). The relative retardance map (Fig. 4c) and optic-axis orientation map (Fig. 4d), on the other hand, provide quantitative visualization of myelin anisotropy, which can be used for general high-resolution imaging of neuroanatomy or for investigating details of myelin pathology in aging or disease. The images presented inFigure 4show a small FOV for visualization purposes; however, our system has the capability to acquire much larger, volumetric tiled image sets of entire rhesus monkey brain sections at high resolution (see SupplementaryFigs. S1 and S2). We can image an entire rhesus monkey brain section (including z-stack acquisition) at 20X magnification (~625 nm optical resolution) in ~18 hours, but this would require ~2 TB of hard drive space. To do this at 40X magnification (~416 nm optical resolution), this would require ~5 times as much data and would take ~5 times longer to acquire. In this work, we have applied these advancements in qBRM to acquire high-resolution, label-free images of myelin structure, for the purpose of characterizing structural changes to individual myelinated axons. In our efforts, we have identified several steps during tissue processing or handling that may induce unwanted structural damage to myelin. These results serve a dual purpose: to highlight how sensitive myelin is to common tissue processing methods and to demonstrate the effectiveness of BRM for visualizing structural alterations to individual myelinated axons. By identifying and characterizing how sensitive myelin lipids are to damage in controlled experiments, it has enabled us to better optimize sample preparation techniques for effective BRM of myelin structure.

### 3.2 Preserving myelin by preventing the tissue from drying on the microscope slide

**Fig. 5.**
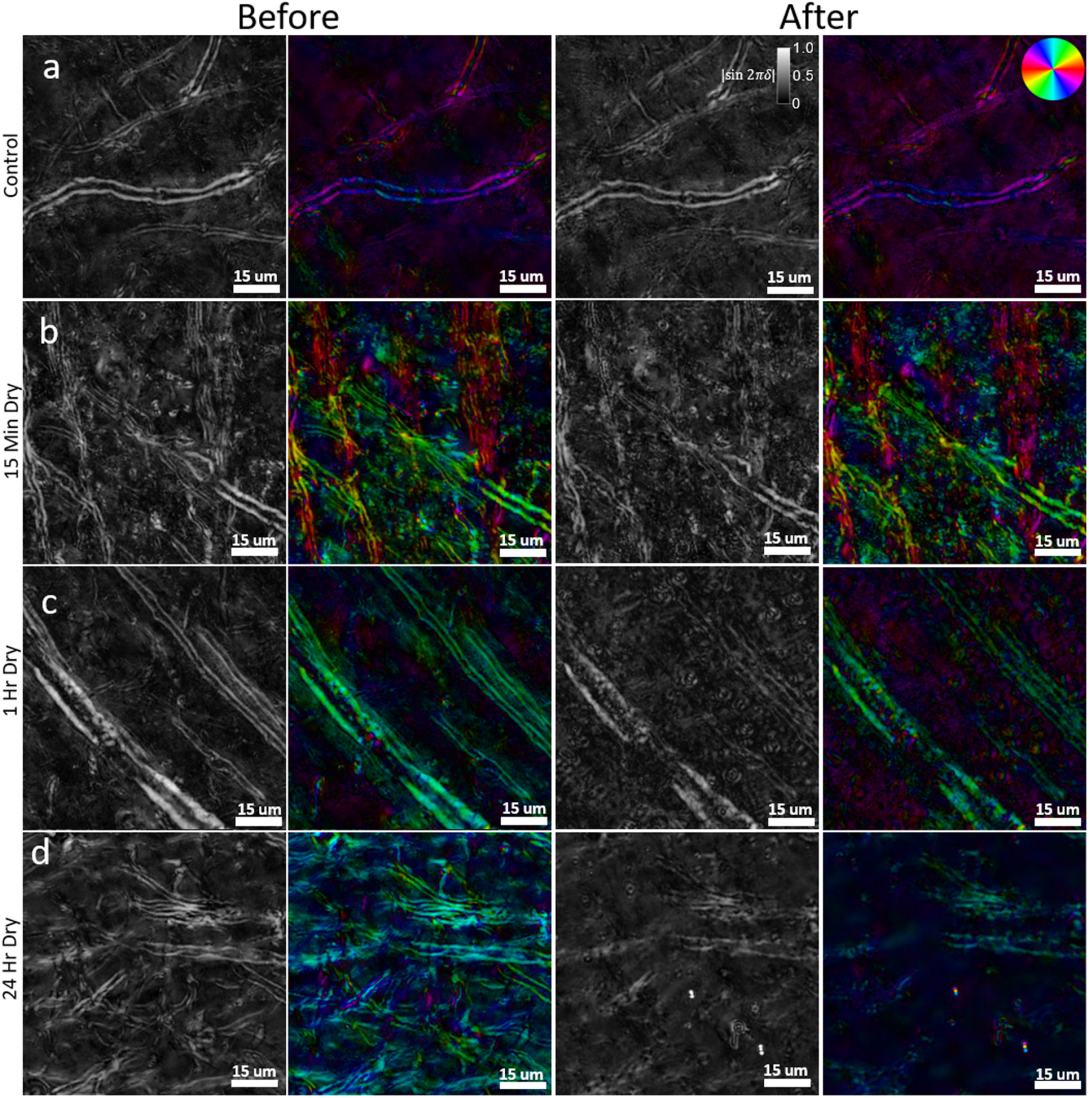
High-resolution (40X, NA 0.75) z-stack qBRM images (focus-stacked) of brain sections that have been allowed to air dry for prolonged amounts of time during microscope slide mounting. In each row, relative retardance and optic-axis orientation images were taken before damage to myelin and after damage to myelin, following prolonged air drying of the sample on the microscope slide. (a) Control, 0 minutes drying. (b) 15 minutes drying. (c) 1 hour drying. (d) 24 hour drying. The orientation of the optic axis is shown by the color wheel in the top-right of (a).

Extended tissue drying is a common sample preparation step in microscopy to ensure adherence of the section to the microscope slide during staining protocols and coverslipping. In tissue sections that were first mounted and imaged following our ideal slide preparation procedure (seeSec. 2.1.2), then subjected to four different experimental drying times (seeSec. 2.1.3), we observed a clear loss of myelin integrity and a decrease in relative retardance in the re-coverslipped and reimaged tissue. Before-and-after z-stack images (focus-stacked) are shown for drying times of 0 minutes (control), 15 minutes, 1 hour, and 24 hours (Fig. 5), and the relative retardance was calculated for several ROIs for each tissue section to demonstrate loss of intact myelin structure (seeSec. 2.2.8). While individual images are presented from a single ROI of one brain section, the images are representative of damage seen through the entire subset of tissue that was subjected to the same drying conditions.

The control sample (Fig. 5a), which underwent the same experimental tissue handling procedures, but was not given extended time to dry, shows no evidence of structural damage to myelin after re-coverslipping and has a marginal average decrease in relative retardance of 3.36%. The small decrease in retardance of the control sample is likely caused by small variations when solving for retardance as well as any tissue handling that could incur slight damage to myelin. After 15 minutes of drying (Fig. 5b), we observed subtle changes to structural integrity of myelin (relative retardance decrease of 15.77%) and the formation of spherical lipid vesicles that are found in the tissue and free floating in the glycerol mounting medium. As myelin degenerates, we observe two general tendencies: reduced relative retardance of intact myelinated axons and increased variety of local optic-axis orientation as degenerated myelin forms vesicular structures. While the changes are relatively subtle at this time point, the level of myelin damage is still readily apparent and shows that myelin damage due to tissue dehydration begins within a matter of minutes. After 1 hour of drying (Fig. 5c), the structural damage to myelinated axons becomes extensive with an average relative retardance decrease of 28.70%, and a much more significant amount of spherical lipid vesicles is found throughout the tissue. The formation of these vesicle structures is consistent with the degradation of myelin, as their edges are thick and exhibit strong birefringence—the same as that of myelin. Structures with single lipid bilayers, such as air bubbles or cells, do not generate the same level of birefringence contrast along their edges. In the case of this sample, as the lipid vesicles are still located within the ROI that was imaged, the myelin breakdown can be seen by a sharp disruption of myelin integrity and an increase in the variety of local optic-axis orientation where the vesicles have formed. With more time or with rinsing of the sample, the lipid vesicles can be cleared from this ROI to show the remaining myelinated axons, which have significantly reduced relative retardance. Similar but more dramatic changes can be seen after 24 hours of drying (Fig. 5d), where there is major structural damage to most of the myelin sheaths in the FOV, and there is a large loss in overall retardance of 29.93%. Throughout the sample, in other ROIs, there is also an extensive accumulation of lipid vesicles. While the lipid vesicles are derived from degenerated myelin in the tissue, we have observed that these lipid vesicles tend to settle at a focal plane outside the tissue, and eventually they coalesce and may form larger aggregates of lipid at the edge of tissue and in blood vessels. As neurodegenerative diseases in the CNS are generally expected to result in a reduction of myelin or axonal density as well as microstructural alterations to individual myelin sheaths, it is vital to ensure that myelin changes quantified by metrics of birefringence are solely due to disease changes and not induced by user error during sample preparation. These results show that drying or dehydration of brain tissue causes structural damage to myelin and must be avoided to ensure structural preservation for high-resolution imaging of myelin with BRM.

### 3.3 Preserving myelin and refractive index-matching by selecting the correct mounting medium

**Fig. 6.**
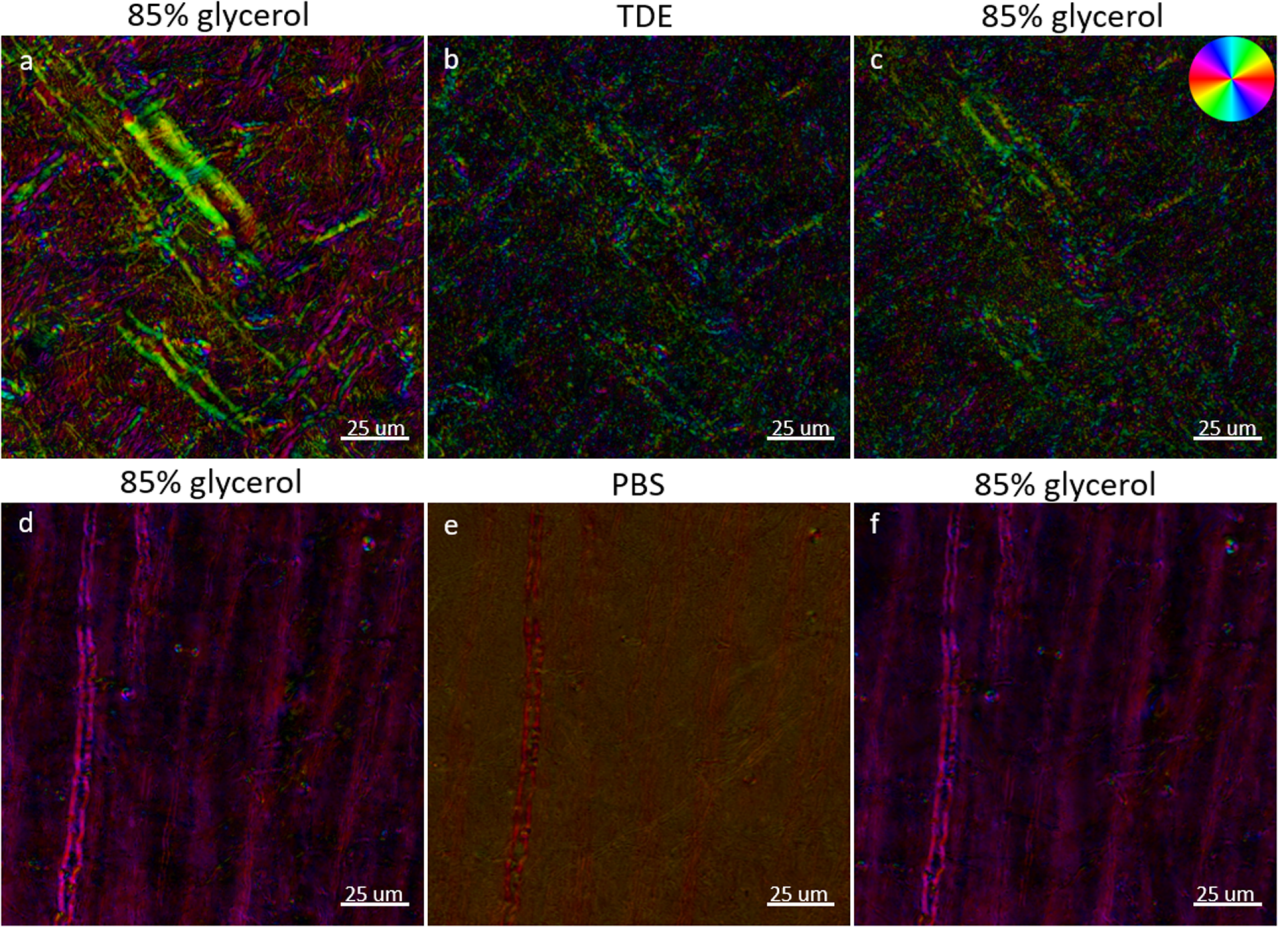
High-resolution retardance weighted optic-axis orientation (40X, NA 0.75) qBRM z-stack images (focus-stacked) of brain sections that have been prepared with two different experimental mounting media. (a, d) Sections were initially mounted in 85% glycerol (n ≈ 1.46) and imaged under ideal conditions. The sections were then washed and re-coverslipped with an experimental mounting medium of (b) 60% TDE (n ≈ 1.45) or (e) PBS (n = 1.33) and imaged in the same ROIs. (c, f) The sections were then washed again, re-coverslipped in 85% glycerol, and imaged in the same ROIs to assess if permanent damage was done to myelin. The orientation of the optic axis is shown by the color wheel in the top-right of (c).

For BRM, proper selection of the mounting medium is vital for preservation of the tissue section and for providing RI-matching to reduce optical scattering, as the ability to accurately extract the optic axis and relative retardance parameters degrades with increased scattering. Since the anisotropic structure of myelin lipids must be preserved for imaging with BRM, reduction of scattering through optical clearing (e.g., CLARITY (Chung et al., 2013;Tomer et al., 2014), DISCO (Renier et al., 2014;Silvestri et al., 2021)) is highly incompatible, as these approaches involve the complete removal of myelin lipids from brain tissue (delipidation). We have explored a variety of traditional mounting or embedding media for brain sections and have established 85% glycerol as our preferred RI-matching medium for myelin imaging with BRM. And 85% glycerol is easy to work with, provides RI-matching to myelin lipids (n ≈ 1.46), and preserves myelin structure. In tissue sections that were first mounted and imaged following our ideal slide preparation procedure (seeSec. 2.1.2), then re-coverslipped and reimaged in two different experimental mounting media (seeSec. 2.1.4), we observe that the incorrect choice of mounting medium can lead to myelin structural degradation or a loss of birefringence contrast during imaging (Fig. 6). TDE (Fig. 6a-c) was investigated to demonstrate the damage that can occur to myelin structure when treating and mounting sections with an optical clearing agent, while PBS (Fig. 6d-f) was investigated to demonstrate the loss of birefringence contrast that results from poor RI-matching alone (and to serve as a control).

In baseline images (Fig. 6a, d), with sections mounted in 85% glycerol, high-resolution details of myelin structure can be viewed with BRM. After removing the coverslip, washing away the glycerol, and subjecting the brain tissue to clearing and mounting with TDE, a profound loss of myelin structure was observed (Fig. 6b). This is consistent with the observation that the ultrastructure of myelin is affected by TDE clearing (Costantini et al., 2015). In addition to the visible structural alterations to myelinated axons, TDE clearing led to the formation of a large amount of free-floating lipid vesicles/debris throughout the sample, similar to what was observed in tissues that have undergone extensive drying (seeSec. 3.2). To verify that the loss of image quality in the TDE-mounted section (Fig. 6b) was due to damage of the structure of myelin, and not related to poor RI-matching, the coverslip was removed once again, the sample was thoroughly washed in PBS, re-coverslipped in 85% glycerol, and reimaged (Fig. 6c). While the PBS wash rinsed away most of the free-floating lipid vesicles that were generated by TDE, the damage to myelinated axons in the FOV was still present after re-coverslipping the tissue in 85% glycerol, confirming that TDE clearing leads to permanent myelin damage. These effects of TDE can be seen as a loss of relative retardance (~54% decrease) across the FOV and a disruption in the local orientation of the myelin optic axis for individual myelinated axons. When performing the same procedure, but with PBS as the experimental mounting medium, hazing and reduced birefringence contrast were observed (Fig. 6e) due to poor RI-matching. The sample was washed in PBS and re-coverslipped in 85% glycerol (Fig. 6f) to show the same structures as the initial baseline image (Fig. 6d), which serves as a negative control and demonstrates that tissue handling (i.e., coverslip removal, rinsing, and flattening tissue) with PBS and glycerol, during slide preparation, does not induce damage to the structure of myelin. The sample showed an increase in retardance of 2.34%, which can be considered within normal variation due to tissue handling (seeSec. 3.2). Given these results, optical clearing or RI-matching with TDE should be avoided for structural imaging of myelin with BRM.

### 3.4 Preserving myelin by avoiding detergents used for membrane permeabilization

**Fig. 7.**
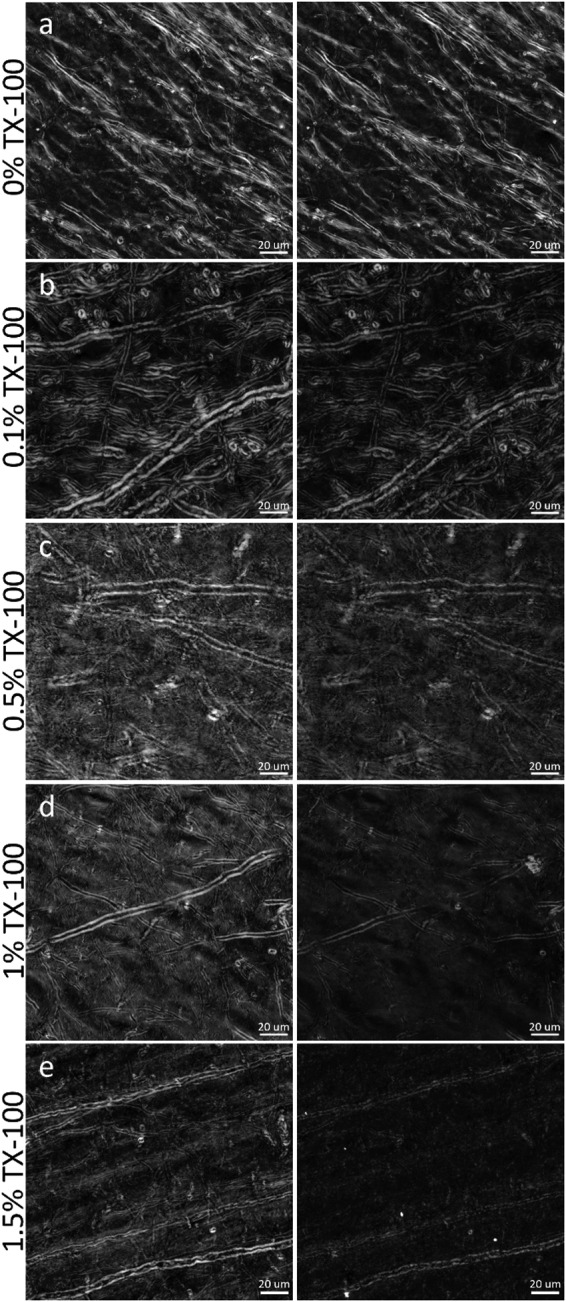
High-resolution (40X, NA 0.75) qBRM z-stack images (focus-stacked) of brain sections that were subjected to different concentrations of TX-100 for characterizing the effects of detergent permeabilization during IHC. (Left) Sections were initially mounted in 85% glycerol and imaged under ideal conditions. (Right) The sections were then subjected to detergent permeabilization by treatment with (a) 0% TX-100 (control), (b) 0.1% TX-100, (c) 0.5% TX-100, (d) 1.0% TX-100, or (e) 1.5% TX-100 and were re-coverslipped with 85% glycerol and imaged again in the same ROIs to assess if damage had been done to myelin. Myelin damage can be visualized by direct alterations to its structure or by a loss of relative retardance.

IHC is an effective method for specific labeling of biological structures in tissue sections. However, to ensure labeling efficiency throughout frozen tissue sections, IHC protocols commonly employ detergents, which act to permeabilize cell membranes for better antibody penetration (Stewart et al., 2018). For IHC labeling of myelin proteins (MBP or PLP), this detergent permeabilization step is particularly important, as myelin proteins are embedded within the lipid layers of the myelin sheath and cannot be easily accessed (Gonsalvez et al., 2019). As the process of labeling MBP or PLP necessitates direct disruption of the lipid structure of myelin, either through detergent permeabilization, optical clearing, or deparaffinization, IHC will likely be limited in its ability to provide a measure of the integrity of the myelin sheath in the same way that is possible with BRM. To demonstrate this, BRM was used to measure the optical anisotropy of myelin lipids after tissue processing for IHC, providing quantitative visualizations of myelin damage as a result of permeabilizing the multilayered membrane structure of myelin. In tissue sections that were first mounted and imaged following our ideal slide preparation procedure (seeSec. 2.1.2), then subjected to a titration of TX-100 concentrations (0%, 0.1%, 0.5%, 1.0%, and 1.5%) to simulate IHC at varying levels of harshness (seeSec. 2.1.5), we demonstrate that permeabilization for IHC leads to a profound loss of myelin anisotropy (relative retardance) and a concentration-dependent disruption to structure (Fig. 7). While individual images are presented from small ROIs, the images are representative of the extent of damage seen throughout the entire tissue section for each concentration of TX-100.

In baseline images, taken before exposing the tissue sections to TX-100, myelin is seen in high-resolution detail with BRM. After removing the coverslip, washing away the glycerol mounting medium, rinsing the tissue in its designated concentration of TX-100, and imaging again, the processed samples showed widespread changes to myelin structure across the tissue section. For every tissue section exposed to TX-100, we observed a reduction of relative retardance (~25–40%) across the FOV, while the control samples (Fig. 7a) remained effectively unchanged (~5%). When qualitatively viewing the images taken after exposure to TX-100, it is clear that there was a further concentration-dependent level of damage to the structure of myelinated axons, which is unsurprising given the established effect of TX-100 on lipid membranes. In the section rinsed with 0.1% TX-100 (Fig. 7b), myelinated axons exhibit less obvious qualitative changes to gross structure, but still exhibit a loss of relative retardance compared with the baseline image. We have also observed that saponin, another common detergent used for IHC permeabilization, has a similarly small, but noticeable effect on myelin integrity (data not shown). In the sections that were exposed to 0.5% TX-100 (Fig. 7c), 1.0% TX-100 (Fig. 7d), and 1.5% TX-100 (Fig. 7e), there was much more extensive myelin degradation observed, which can be seen by a progressive decrease in the relative retardance across each FOV, reaching the point where smaller myelinated axons drop below detectable levels of birefringence contrast or are completely destroyed (Fig. 7d, e). These results are largely expected and provide evidence that permeabilization with TX-100 causes a loss of myelin content in the form of reduced myelin anisotropy (relative retardance), even at low concentrations.

While studies may benefit from both BRM, for characterization of myelin lipids, and IHC, for either cross-validation of BRM or for characterization of other structures such as cells, axons, or blood vessels, these results establish that these techniques are fundamentally incompatible in the same brain sections. If absolutely desired, it may still be possible to sacrifice BRM contrast to perform a minimally harsh permeabilization for IHC; however, weak permeabilization likely will not be effective for achieving good antibody penetration into myelin, to label MBP or PLP. If planning a study that utilizes both BRM for imaging of myelin and IHC for imaging of myelin proteins or other structures, imaging with BRM should, ideally, be performed prior to membrane permeabilization and tissue processing for IHC.

### 3.5 Imaging details of individual myelinated axons by selecting an optimal tissue-section thickness

**Fig. 8.**
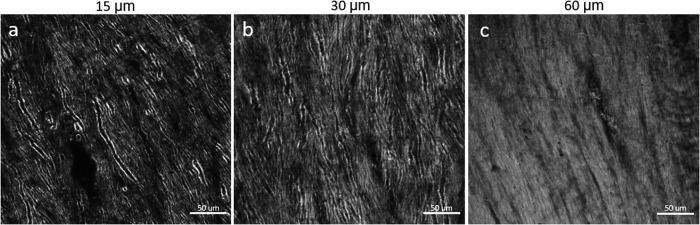
High-resolution 40X (NA 0.75) CCP-BRM z-stack images (focus-stacked) of the corpus callosum sectioned at thicknesses of (a) 15 µm, (b) 30 µm, and (c) 60 µm.

Section thickness is an important consideration for BRM imaging of myelin, depending on the specific aim of a study. In general, imaging myelinated axons at high resolution is limited to sections <100 µm to preserve birefringence contrast during transmission of polarized light through the tissue. Scattering and out-of-focus birefringence limit the ability to resolve individual myelin sheaths in thicker sections. To assess imaging quality as a function of section thickness, three sections taken from the same rhesus monkey brain were cut at 15 µm, 30 µm, and 60 µm (seeSec. 2.1.6) and compared in white matter (Fig. 8) and gray matter (see “Sample_thickness/Gray_matter” in (Gray & Blanke, 2023)) based on empirical observations. Thinner sections (<30 µm) offer the ability to resolve myelin structure at the single-axon level, even in very dense white matter tracts, such as the corpus callosum (Fig. 8a, b).

In thicker sections, with more myelinated axons being imaged along the z-dimension, there are hazing effects from the myelinated axons outside of the focal plane being imaged. In the 60-µm section of corpus callosum (Fig. 8c), it is much more difficult to resolve the details of individual axons compared with the corpus callosum sectioned at 15 µm (Fig. 8a) and 30 µm (Fig. 8b). While thinner sections are generally preferable for imaging white matter, in more sparsely myelinated gray matter regions, thicker sections may enable better volumetric imaging of individual axons (refer to “Sample_thickness/Gray_matter” in (Gray & Blanke, 2023)). Thin sections can also be used for investigating gray matter regions but at the loss of some volumetric information. With all things taken into account, our preferred section thickness for high-resolution BRM is 30 µm, as that maintains good contrast during imaging and is reliable to cut and mount on microscope slides.

## 4 Discussion

### 4.1 Summary

This work describes the design of a new BRM system optimized for high-throughput, label-free imaging of myelin in the rhesus monkey brain at submicron resolution (down to ~250 nm). This system allows for rapid visualization and quantitative analysis of myelin structure at multiple scales across entire brain sections, to enable large-scale studies of myelin breakdown in aging, injury, and disease. To properly assess the role that myelin degeneration plays in disease contexts, myelin structure must be adequately preserved during the preparation of the tissue for imaging. With our BRM system, we have demonstrated that myelin integrity at high resolution is adversely affected by several common tissue processing techniques that either cause direct damage to myelin structure or cause a reduction in image quality. For effective BRM of myelin, we have shown that (1) tissue must be prevented from drying on the microscope slide, (2) the correct mounting medium must be selected, (3) detergent permeabilization must be avoided, and (4) the optimal tissue section thickness must be selected based on the region of interest. Although our work focuses on optimizing structural imaging of myelin with label-free BRM, the mechanisms of myelin degradation that we have demonstrated raise concerns about the ability to accurately image and quantify myelin structure with other methods that require extensive processing of tissue (e.g., IHC of myelin proteins). As we have observed the tendency for myelin lipids to dissociate from axons and fully extract from the tissue section following inadequate handling and preparation of tissue, these results also suggest that unanticipated structural damage to myelin during sample preparation could be a significant source of variability in other label-free imaging approaches, as well as during traditional histochemical staining of myelin.

### 4.2 Goals of Myelin Imaging

There are two primary motivations for structural imaging of myelin within postmortem brain tissues: (1) to study trajectories of myelinated axons for neuroanatomy and (2) to study structural alterations to myelin and their relationship with functional deficits in disease. For studying bundles of myelinated axons in white matter, diffusion-weighted magnetic resonance imaging (dMRI) has emerged as a powerful technique for volumetric imaging both in vivo and ex vivo (Schmahmann et al., 2007;Wedeen et al., 2008). However, as dMRI is limited to hundreds of microns in resolution, it cannot determine trajectories of individual myelinated axons or identify the development of myelin pathology of single axons. To address these shortcomings, other groups performing myelin microscopy have largely focused on utilizing myelin as a means of quantifying orientations of axons at more mesoscopic resolution, to serve as validation of dMRI data (Axer & Amunts, 2022;Budde & Frank, 2012;Khan et al., 2015;Menzel et al., 2021;Mollink et al., 2017;Ronen et al., 2014;Schilling et al., 2018;Sorelli et al., 2023), rather than optimizing imaging for the microscopic assessment of myelin integrity at the level of individual axons. As BRM can be carried out with any objective lens, there is ultimate flexibility to image brain tissue at any desired optical resolution, enabling low-resolution imaging of white matter trajectories across entire brain sections as well as high-resolution imaging of individual myelinated axons. This multiscale imaging capacity adds to the utility of BRM, as the resolution and size of volumetric image acquisitions can be effortlessly tuned depending on the application.

For studying pathological changes to myelinated axons in disease, higher-resolution imaging of myelin structure is typically required. While large-scale loss of myelin is seen in demyelinating diseases or advanced neurodegeneration, which may be quantifiable with label-based or low-resolution myelin imaging methods, the more subtle instances of myelin pathology, at earlier disease stages, would likely not be captured by these techniques. For instance, with EM, subtle myelin pathology has been observed in aging, where degradation is characterized by structural changes to individual myelin internodes at the microscopic or ultramicroscopic levels (Bowley et al., 2010;Feldman & Peters, 1998;Peters, 2009;Peters & Sethares, 2002). Historically, high-resolution imaging of myelin pathology has been restricted to these EM studies, even though structural alterations to myelin can reach ~1 µm in size or larger, which is well within the resolution capabilities of diffraction-limited optical microscopy. To this end, we have previously shown the capability of BRM to image myelin debris in a rhesus monkey model of cortical injury (Blanke et al., 2021). In our work moving forward, we aim to investigate and validate the imaging of subtle myelin pathology that has been shown to occur in aging and other age-related neurodegenerative diseases.

### 4.3 Other Optical Microscopy Methods for Imaging Myelin

Other microscopy techniques that have demonstrated sensitivity to myelin degeneration are CARS microscopy (Canta et al., 2016;Gasecka et al., 2017;Ozsvár et al., 2018;Wang et al., 2005) and SCoRe microscopy (Gonsalvez et al., 2019;Hill et al., 2018;Schain et al., 2014). CARS is a nonlinear optical microscopy technique that targets specific vibrational modes of the lipid molecules that make up myelin, providing high sensitivity and specificity for imaging of individual myelinated axons. Since CARS has intrinsic optical sectioning capabilities, it is a powerful method for high-resolution imaging. Similarly, SCoRe is another high-resolution myelin imaging method that provides optical sectioning, wherein contrast to myelin is generated by confocal detection of spectrally reflected light at the interface between axons and the surrounding layers of myelin. While these techniques image in a reflectance geometry and, therefore, have the potential to be used for in vivo characterization of changes to myelin over time, in vivo imaging of myelin is fundamentally limited to peripheral nerves or the most superficial layers of cortex. For assessment of myelin pathology in postmortem tissues, these label-free approaches, in addition to label-based approaches that utilize standard confocal fluorescence microscopy, are expensive, time-consuming, and significantly less efficient than BRM, as they utilize point-scanning to form an image and are limited to small fields of view. Compared with many other forms of microscopy, our implementation of BRM is an inexpensive technique with a low barrier to entry. Even for the most inexperienced microscopists, a simple CCP-BRM setup can be configured for real-time, qualitative imaging of myelin, without requiring substantial design work or system modifications (seeSupplementary Note 2.3).

### 4.4 Limitations of BRM

Although BRM provides adequate optical resolution for the detection of even the smallest-diameter (~0.2–0.3 µm) myelinated axons, there are practical limitations imposed by the thickness of the sections, which prevent sensitive imaging of this population of fibers. As the optical birefringence of myelin is destroyed by the process of embedding in standard resins or plastics, ultrathin sections cannot be generated to perform BRM in the same way that it is done for myelin imaging with EM or other staining techniques. Thus far, we have successfully generated cryostat sections of smaller-area tissues at thicknesses of 10–15 µm, but for whole-brain sections of rhesus monkeys, we have largely been working with 30-µm-thick sections. Since the tissue sections for BRM have a minimum thickness that far exceeds the diameter of a typical fiber, and because most of the myelin that we are interested in assessing is located within dense white matter, we have limited sensitivity to image smaller myelinated axons and detect subtle myelin pathology in these regions. In superficial white matter and gray matter, with lower densities of myelinated axons, detailed imaging of individual myelinated axons can be performed much more reliably. In future work, reliable methods for generating thinner sections will likely lead to much more informative imaging of myelin structure within white matter. Another practical limitation of BRM for the study of myelin pathology, which also impacts other myelin imaging techniques, is the postmortem interval (PMI) specific to human brain tissues. It has been reported that PMI should be limited to <24 hours for preservation of myelin integrity in human tissues (Axer et al., 2011;Shepherd et al., 2009), and, in general, minimizing PMI would be the ideal case if the goal is to characterize subtle changes to myelin in disease. In addition to these considerations about PMI, the type of fixation can also play a critical role in the quality of the tissue and the integrity of the myelin. In the rhesus monkeys, perfusion-fixation provides a much more even degree of fixation across the brain than what is possible with human tissues that must be fixed by immersion. In immersion-fixed tissues, the outer layers of the brain are fixed more rapidly than the inner layers, leading to differential fixation that could result in a high degree of variability in myelin integrity across the tissue. These factors related to postmortem effects in immersion-fixed human tissues complicate the characterization of myelinated axons at high resolution and should be taken into consideration for utilizing BRM on immersion-fixed tissues.

## 5 Conclusions

We have developed a new BRM system that enables efficient, high-throughput imaging of myelin in postmortem brain sections. In our work, we have advanced BRM for the study of myelin degeneration in the context of disease. We have demonstrated that myelin is highly susceptible to unintentional breakdown during inadequate handling of tissue or from standard IHC processing methods, and we have used this knowledge to optimize sample preparation for more effective myelin imaging with BRM. The experiments carried out in this study provide unequivocal evidence that harsh or extraneous tissue processing steps should be avoided when imaging myelin structure and assessing features of myelin pathology. As such, when integrating BRM into other studies, BRM should be used to image myelin structure first, before subjecting tissues to processing for IHC or other stains. By following this workflow, label-free BRM can provide valuable insights into the disease-related degradation of myelin, while still preserving the same tissue sections for further processing. As label-free BRM provides a method for direct imaging of myelin based on structural contrast, we anticipate that BRM will be an effective method for large-scale (and potentially automated) quantification of structural alterations to myelin across collections of brain sections. Since degeneration or loss of myelinated axons likely plays a key role in the manifestation of numerous diseases, quantitative assessment of myelin structure with BRM may offer new insights into disease progression and the efficacy of treatments that look to limit damage or enhance repair of brain tissue. Based on its capabilities for novel, high-resolution imaging of myelin structure with a simple light microscope, BRM should find widespread use in neuroscience for studying myelin in various applications and diseases.

## Data and Code Availability

All data and supplementary material are included in an online repository (Gray & Blanke, 2024) DOI: 10.17632/m2k69b6ftz.3. In addition, all uncropped images taken (not included in the manuscript) of experimental tissue are available upon request. All code for motor control, image acquisition, and qBRM processing has been made available here:https://github.com/alexgray103/BRMscope/.

## Author Contributions

Alexander J. Gray: Conceptualization, Data Curation, Methodology, Software, Writing—Original Draft, Writing— Review and Editing, Visualization, Validation, Investigation. Nathan Blanke: Conceptualization, Methodology, Writing—Original Draft, Writing—Review and Editing, Validation, Investigation. Rhiannon E. Robinson: Investigation, Resources, Writing—Original Draft. Anna Novoseltseva: Software, Validation, Methodology. Douglas L. Rosene: Supervision, Writing—Review and Editing, Funding Acquisition. Irving J. Bigio: Supervision, Writing—Review and Editing, Funding Acquisition.

## Funding

The authors acknowledge funding from the U.S. National Institutes of Health (R01 – AG075727, R01 – AG075876, R01 – AG078460, R21 – NS111174, R21 – NS102991, RF1 – AG062831, RF1 – AG043640), the U.S. National Science Foundation (DGE-1633516), and the Boston University Office of Research – Kilachand Fund.

## Declaration of Competing Interest

The authors declare no competing interests.

## Supplementary Materials

Supplementary material for this article is available with the online version here:https://doi.org/10.1162/imag_a_00186.

## Supplementary Material

Supplementary Material
