# Supplementary material for: Practical considerations for birefringence microscopy of myelin structure: Microscope design and tissue processing for effective imaging
Source: Imaging Neurosci (Camb). Author manuscript; Available in PMC 2025 Jul 29. (PMC12247593; doi:10.1162/imag_a_00186)
Supplement: Supplementary material [file NIHMS2029801-supplement-Supplementary_material.pdf]

Practical considerations for birefringence microscopy of myelin structure: Microscope design and tissue processing for effective imaging – Supplementary Material

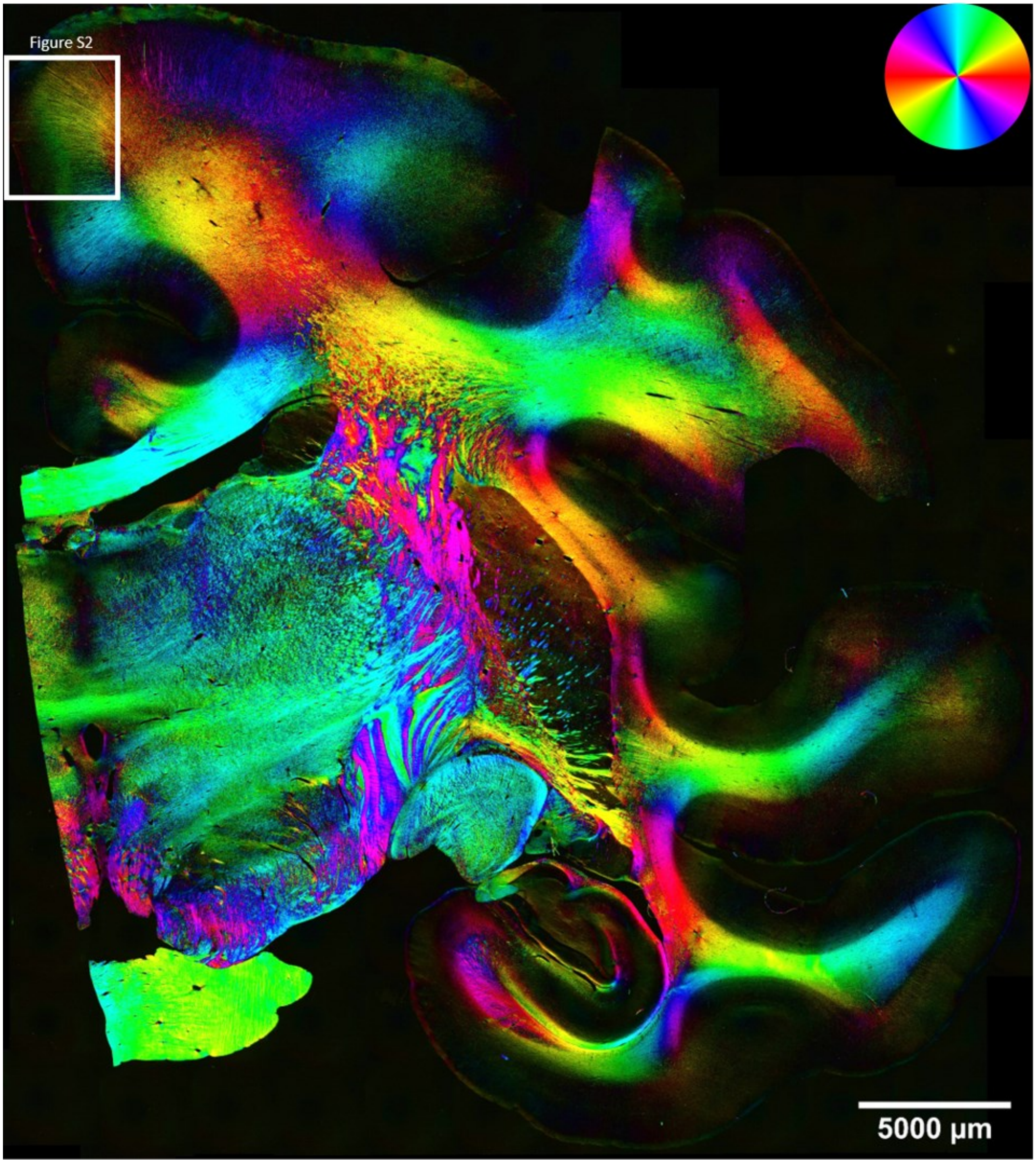

Fig. S1: Low-resolution (4X, NA 0.13) tiled image set of a rhesus monkey coronal brain section (one hemisphere) visualized as a qBRM retardance-weighted optic-axis orientation map. The color wheel in the top-right corner indicates the direction of the myelin optic axis. The white box indicates an area that was imaged at higher resolution (20X, NA 0.5) (See Fig. S2). For a high-resolution version of this image, please refer to (Gray & Blanke, 2023).

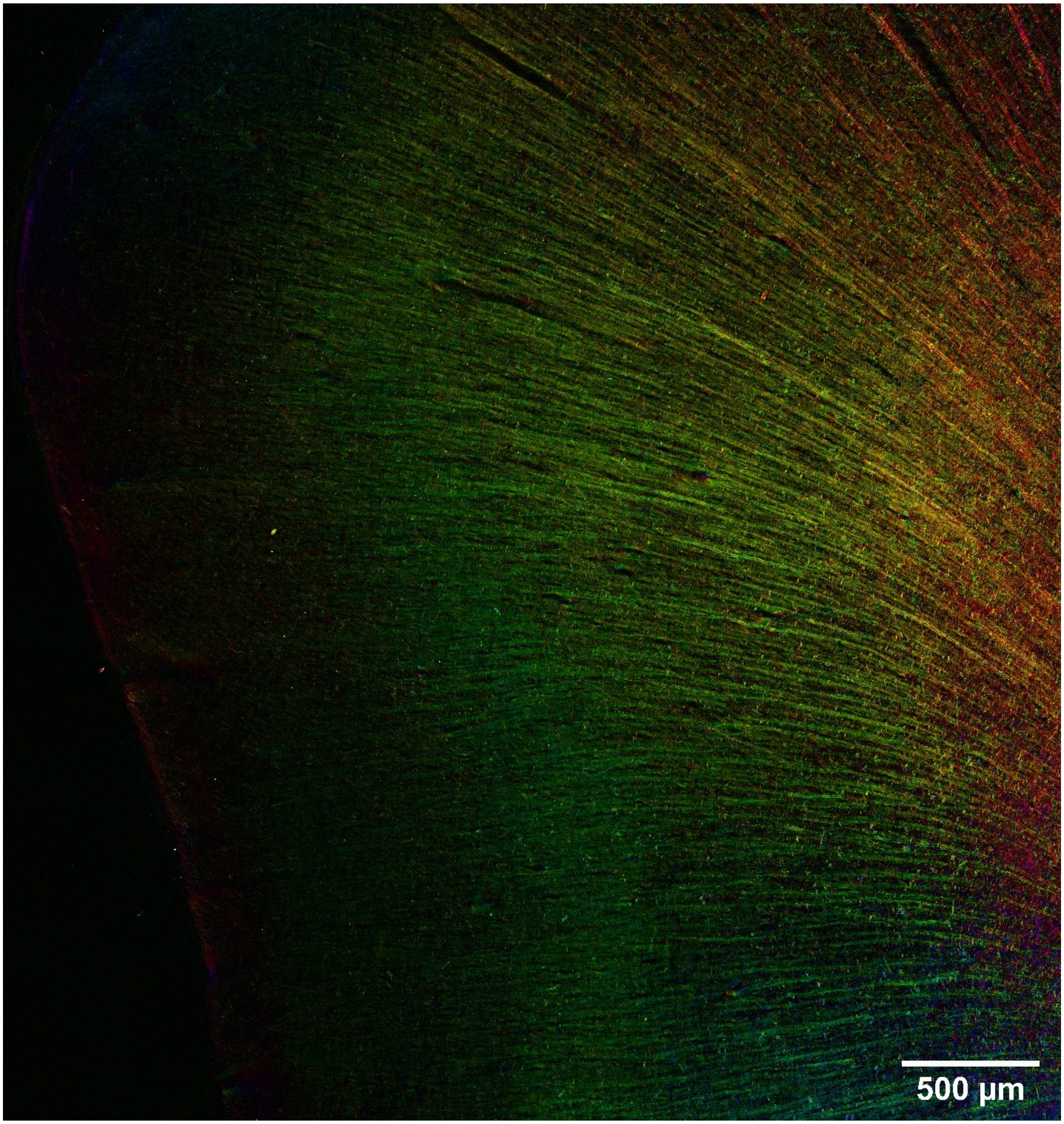

Fig. S2: High-resolution (20X, NA 0.5) tiled image set of a rhesus monkey coronal brain section visualized as a qBRM retardance-weighted optic-axis orientation map. The color wheel for this image can be found in Fig. S1 and indicated the myelin optic axis. For a high-resolution version of this image, please refer to (Gray & Blanke, 2023).

Supplementary Note 1: Unprocessed qBRM visualization

This method for visualization of qBRM data involves saving the three unprocessed qBRM images into an RGB TIFF file, which provides both an efficient method for storing the data as well as a novel and interesting method for viewing the data as a qualitative optic-axis orientation map without processing. During qBRM acquisitions, the images are taken at 0°, 60°, and 120°, which provides a uniform sampling of the sinusoidal signal described in Eq. 1. In areas of low retardance (i.e., gray matter), Eq. 1 approaches $\frac{I_0}{2}$, providing transmittance contrast to the qualitative orientation map such that scattering structures such as cells or blood vessels are darker than the rest of the image. In areas of higher retardance and/or low transmittance (i.e., white matter), the weighting for the qualitative orientation map becomes a complex combination of both retardance and transmittance, with bright retardance contrast being dominant over the dark transmittance contrast. For pixels that contain birefringent myelin of some orientation, the color is blended based on the linear combination of intensities for the RGB (0°, 60°, 120°) color channels. For pixels with an optic axis oriented at -45° (relative to the circular analyzer) their intensity will be highest in the red channel, while pixels with an optic axis of 15° and 75° will be highest in the green and blue channels, respectively. Mathematically, the intensity from Eq. 1 reaches a maximum value when phi equals -45° and the sine term goes to 1. It should be noted, however, that this qualitative optic-axis orientation map has a colormap that is different compared to the optic-axis orientation maps obtained from qBRM processing, due to how the sinusoidal signal is sampled in raw RGB space.

Supplementary Note 2: Methods for Implementation of BRM

For users interested in BRM, there are generally three levels of implementation with different degrees of complexity and instrumentation requirements. Before implementing a complex BRM system, many users will likely be interested in testing their imaging capabilities with a simple setup.

2.1 Custom-built BRM system (Most complex)

This involves assembling a custom BRM system from off-the-shelf parts and optics, such as the design in this paper, for the purposes of carrying out maximally efficient birefringence imaging with both CCP-BRM and qBRM. The system is built from scratch to allow full customization of the optical path and to avoid space restraints imposed by the integration of a BRM system into an existing microscope. A fully capable CCP/qBRM system will require XYZ translational stages as well as rotational stages for fully automated acquisition. As all stages are purchased from third-party sources that provide simple integration into commonly used software packages (e.g., MATLAB & LabVIEW), microscope and camera control can be fully integrated into one program.

2.2 CCP-BRM/qBRM integration into a commercial microscope (Less complex)

A simpler implementation of a BRM system, which does not require fully designing a custom microscope from scratch, is to integrate motorized rotational stages onto any commercial microscope using custom mounts. With rotational stages, the fundamental aspects of CCP-BRM and qBRM can be easily carried out, while utilizing the existing microscope chassis and XY & Z stages. Many commercial microscopes have enough room to allow for small rotational stages to

be inserted into the optical path with simple 3D-printed or machined mounts. In our first BRM system that was designed around the Olympus IX81 chassis (Blanke et al., 2021), we had to remove the turret that housed fluorescence filter cubes in order to make room for polarization optics (mounted in motorized rotational stages) in the detection-side of the microscope. This implementation of BRM requires modest design work, and since all stages may not be motorized or easily controllable through the same software, there may be additional workarounds required. While potentially less automated and flexible than the most complex implementation, designing a BRM system around an existing microscope can be a good alternative for performing high-quality BRM acquisitions.

2.3 CCP-BRM integration to a commercial microscope (Least complex)

The simplest implementation of BRM, which can be integrated into virtually any transmission microscope, involves the use of off-the-shelf circular polarizers for *only* performing CCP-BRM. As circular polarizers are commercially available and low-profile, they can easily be inserted into filter slots or small gaps along the optical path, without requiring substantial design work or system modifications. For this implementation, two circular polarizers of opposite handedness (one left-handed and one right-handed) are required (e.g., Edmund Optics CP42HE & CP42HER), with one placed in the illumination path and the other placed in the detection path of the microscope. Note that these circular polarizers are designed for green light (550 nm) but can be used to produce approximately circularly polarized light over the entire visible spectrum (400-700 nm). However, since circular polarizers are designed for a single wavelength, the ideal solution is to either use a narrowband LED or a bandpass-filtered lamp for illumination at the specified wavelength of the circular polarizers. With a light source of significant bandwidth, circular polarizers must be rotated with respect to one another to achieve maximum contrast (extinction). While this implementation of BRM only provides the ability to image myelin structure qualitatively with CCP-BRM, it is a very simple and cost-effective technique that is highly accessible to all researchers.

Supplementary Note 3: Error Analysis of solving qBRM with 3 points vs. 18 points

To assess the accuracy of our derived 3-point solution, we have compared the error associated with determination of relative retardance, optic-axis orientation and transmittance relative to a 6-point and an 18-point solution. To perform the analysis, a qBRM image set was taken with 18 points, and birefringence parameters were solved with either all 18 points (evenly spaced by 10°) or 6 points (evenly spaced by 30°) using discrete harmonic Fourier analysis, or with 3 points (evenly spaced by 60°) using the direct solutions provided in Eq. 2 – 4. The percent error for each pixel of the relative retardance and transmittance maps was calculated for 18 vs. 6 points and 18 vs. 3 points using the following equation:

$$\frac{|X_{18} - X_{3,6}|}{X_{18}} * 100\%$$

where X_{18} is the value of relative retardance (or transmittance) solved using 18 points and $X_{3,6}$ is the value of relative retardance (or transmittance) solved using 3 or 6 points. The error in optic-axis orientation (φ) was calculated as the absolute difference between the angle solved with 18 points and 3 or 6 points (Fig. S3).

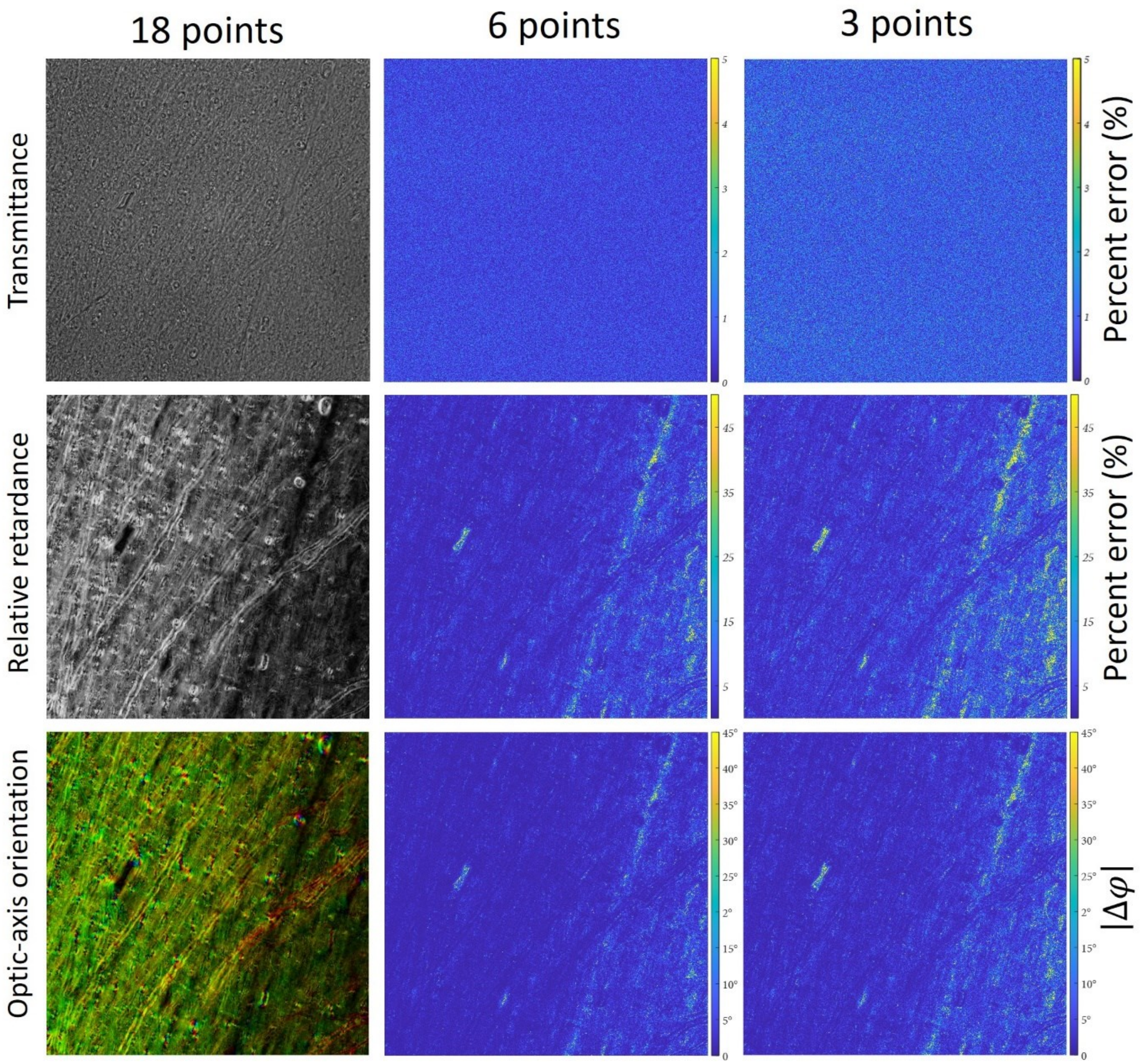

Fig. S3: Error analysis for the fitting accuracy of 18 points with 6 points and 3 points for parameters solve with qBRM imaging (transmittance, relative retardance, and optic axis orientation). For each pixel in the image shown in the left column, the percent error was calculated between the values solved for with 18 points vs. (middle) 6 points and (right) 3 points respectively. The absolute difference of the phi value is shown comparing (middle) 18 points- 6 points and (right) 18 points v. 3 points.

As expected, the accuracy of solving for the 3 birefringence parameters slightly degrades when solving with only 3 points. For pixels with high birefringence (myelinated axons), the 6-point fit and 3-point solution for transmittance have a mean error of 0.26% and 0.41%, respectively. This error increases to 0.6% (6-point) and 0.79% (3-point) in areas that have low or no retardance (no myelinated axons). Similarly, the error when solving for relative retardance is quite low in areas of high retardance signal, with a mean percent error of 1.19% when solving with 3 points and 0.72% when solving with 6 points. Accordingly, in areas of low retardance, this error increases to 4.06% and 6.53% for the 6-point fit and 3-point solution, respectively. The determination of optic-axis orientation follows a similar trend, where for areas of high birefringence, the average difference for the 18 vs. 6-point and 18 vs. 3-point is 0.43° and 0.65°, respectively. For areas of low retardance, the optic-axis orientation difference increases to 2.29° for 18 vs. 6-point and 3.53°

for 18 vs. 3-point. This demonstrates that in areas of little or no birefringence, the ability to accurately extract transmittance, retardance and optic axis orientation with our 3-point solution starts to degrade. In practice, this simply leads to greater noise in pixels that do not contain birefringent structures, which would likely not affect quantitative analysis of myelinated axons with BRM.

Supplementary Note 4: Focus Stacking with Adobe Photoshop

In this work, focus stacking is used for visualization of all high-resolution images acquired as Z-stacks. While there is free software for focus stacking included in Fiji (Forster et al., 2004) or MATLAB (Pertuz et al., 2013), we have found that Adobe Photoshop produces higher quality focus-stacked images with less error. In Adobe Photoshop, focus stacking was performed with the following steps:

1. In FIJI or MATLAB, save the Z-stack as individual images (image sequence).
2. Open Adobe Photoshop
3. "File" → "Scripts" → "Load Files into Stack…"
4. Navigate to the Z-stack saved as individual images and select all images.
5. "Select" → "All Layers"
6. "Edit" → "Auto-Blend Layers…" → "Stack Images"

References

Blanke, N., Go, V., Rosene, D. L., & Bigio, I. J. (2021). Quantitative birefringence microscopy for imaging the structural integrity of CNS myelin following circumscribed cortical injury in the rhesus monkey. *Neurophotonics*, *8*(1), 1–15. https://doi.org/10.1117/1.NPh.8.1.015010

Forster, B., Van De Ville, D., Berent, J., Sage, D., & Unser, M. (2004). Complex wavelets for extended depth-of-field: A new method for the fusion of multichannel microscopy images. *Microscopy Research and Technique*, *65*(1–2), 33–42. https://doi.org/10.1002/jemt.20092

Gray, A., & Blanke, N. (2023). Supplementary Data for “Practical considerations for birefringence microscopy of myelin structure: Microscope design and tissue processing for effective imaging.” In *Mendeley Data, V2*. https://doi.org/10.17632/m2k69b6ftz.2

Pertuz, S., Puig, D., Garcia, M. A., & Fusiello, A. (2013). Generation of All-in-Focus Images by Noise-Robust Selective Fusion of Limited Depth-of-Field Images. *IEEE Transactions on Image Processing*, *22*(3), 1242–1251.
